# Expression of the Intracellular COPT3-Mediated Cu Transport Is Temporally Regulated by the TCP16 Transcription Factor

**DOI:** 10.3389/fpls.2018.00910

**Published:** 2018-07-03

**Authors:** Nuria Andrés-Colás, Angela Carrió-Seguí, Salah E. Abdel-Ghany, Marinus Pilon, Lola Peñarrubia

**Affiliations:** ^1^Departament de Bioquímica i Biologia Molecular, Estructura de Recerca Interdisciplinar en Biotecnologia i Biomedicina, Universitat de València, Valencia, Spain; ^2^Department of Biology, Colorado State University, Fort Collins, CO, United States

**Keywords:** copper transport, COPT3, heavy metals, TCP16, transcriptional regulation

## Abstract

Copper is an essential element in plants. When scarce, copper is acquired from extracellular environment or remobilized from intracellular sites, through members of the high affinity copper transporters family COPT located at the plasma membrane and internal membrane, respectively. Here, we show that COPT3 is an intracellular copper transporter, located at a compartment of the secretory pathway, that is mainly expressed in pollen grains and vascular bundles. Contrary to the COPT1 plasma membrane member, the expression of the internal *COPT3* membrane transporter was higher at 12 h than at 0 h of a neutral photoperiod day under copper deficiency. The screening of a library of conditionally overexpressed transcription factors implicated members of the TCP family in the COPT3 differential temporal expression pattern. Particularly, *in vitro*, TCP16 was found to bind to the *COPT3* promoter and down-regulated its expression. Accordingly, *TCP16* was mainly expressed at 0 h under copper deficiency and induced at 12 h by copper excess. Moreover, *TCP16* overexpression resulted in increased sensitivity to copper deficiency, whereas the *tcp16* mutant was sensitive to copper excess. Both copper content and the expression of particular copper status markers were altered in plants with modified levels of *TCP16.* Consistent with TCP16 affecting pollen development, the lack of COPT3 function led to altered pollen morphology. Furthermore, analysis of *copt3* and *COPT3* overexpressing plants revealed that COPT3 function exerted a negative effect on *TCP16* expression. Taken together, these results suggest a differential daily regulation of copper uptake depending on the external and internal copper pools, in which TCP16 inhibits copper remobilization at dawn through repression of intracellular transporters.

## Introduction

Copper (Cu) is an essential micronutrient for the growth and development of aerobic organisms. Under metal deficiency, Cu^+^ is incorporated through high affinity COPper Transporters, denoted COPTs in plants (Kampfenkel et al., 1995; Sancenón et al., 2003; Puig, 2014; Peñarrubia et al., 2015) and referred to as CTR (SLC31) in other organisms (Kim et al., 2013). In *Arabidopsis thaliana*, the COPT family can be divided into two subfamilies: the plasma membrane members COPT1, COPT2, and COPT6 (pmCOPT), which are transcriptionally induced under Cu deficiency (Sancenón et al., 2004; Garcia-Molina et al., 2013; Perea-García et al., 2013), and the members located in internal membranes COPT3 and COPT5 (imCOPT), which are not clearly induced by Cu deficiency (Sancenón et al., 2003; Garcia-Molina et al., 2011; Klaumann et al., 2011). This subdivision might distinguish at least two Cu^+^ sources in the cells (external and internal), differentially mobilized based on the type of COPT activated.

The transcriptional response to Cu deficiency is mainly orchestrated by the transcription factor (TF) SQUAMOSA PROMOTER BINDING PROTEIN-LIKE7 (SPL7) through binding to GTAC motifs in the promoters of target genes, such as *pmCOPT* (Yamasaki et al., 2009; Bernal et al., 2012). Thus, pmCOPT-mediated cytosolic Cu^+^ uptake from the extracellular environment is highly increased by SPL7 under Cu deficiency. It has been suggested that the SPL7-mediated auto-regulatory effect of Cu on *pmCOPT* expression could establish a feedback loop responsible for cyclic expression, peaking at dawn (Andrés-Colás et al., 2010; Peñarrubia et al., 2010). Deregulated Cu^+^ uptake in COPT-overexpressing plants causes abnormal development in the absence of environmental cycles (Andrés-Colás et al., 2010; Perea-García et al., 2016a,b). Furthermore, the observed interaction between SPL7 and ELONGATED HYPOCOTYL5 (HY5) underscores a connection between Cu homeostasis and light (Zhang et al., 2014).

In yeast, it has been shown that, due to the Cu^+^ toxicity, practically no free Cu^+^ is found in the cytosolic compartment (Rae et al., 1999). Therefore, pmCOPT-mediated Cu^+^ uptake is probably coupled to cupro-chaperone-mediated delivery to different protein targets, among them the P-type ATPase RESPONSIVE-TO-ANTAGONIST1 (RAN1) located at the endoplasmic reticulum (ER) where it pumps Cu^+^ into the lumen (Hirayama et al., 1999). SPL7 presents an operative transmembrane domain that allow his localization at the endomembrane system, most likely the ER. During ER stress, as a result of Cu deficiency, SPL7 localizes in the nucleus to modulate the Cu deficiency responses, after activation by proteolytic cleavage. In this sense, SPL7 could function as a double Cu sensor in both the nucleo-cytoplasm and the ER lumen (Garcia-Molina et al., 2014).

With regard to the imCOPT-mediated Cu^+^ transport from internal sources, COPT5 plays an important role in the plant response to severe environmental Cu scarcity (Garcia-Molina et al., 2011; Klaumann et al., 2011). COPT5 functions in remobilizing Cu from prevacuolar vesicles, which could act as internal stores or recycling vesicles to provide the metal to key Cu-dependent processes such as photosynthesis (Garcia-Molina et al., 2011; Klaumann et al., 2011; Carrió-Seguí et al., 2015). Little is known about the function of COPT3. COPT3 has been classified as a member of the imCOPT subfamily based on its partial complementation of the respiratory and Cu transport defect exhibited by a *ctr1Δctr3Δ* yeast mutant (Sancenón et al., 2003; Garcia-Molina et al., 2013). Apparently, *imCOPT* expression is not affected by Cu and temporal factors modulating *imCOPT* expression remain unexplored.

The TCP (named after TEOSINTE BRANCHED 1, CYCLOIDEA and PROLIFERATING CELL FACTOR 1) protein family precisely orchestrates spatial and temporal plant responses to both environmental and endogenous factors (Martín-Trillo and Cubas, 2010; Kieffer et al., 2011; Li, 2015; Danisman, 2016; Dhaka et al., 2017). The TCP family is constituted by plant-specific TFs that share a conserved non-canonical basic helix–loop–helix (bHLH) DNA binding domain, termed TCP domain (Cubas et al., 1999). The TCP members, are grouped into two subfamilies, based on the TCP domain structure and their roles (Cubas et al., 1999). These subfamilies are denoted as class I or PROLIFERATING CELL FACTOR (PCF) and class II, which is divided in CINCINNATA (CIN) and CYCLOIDEA (CYC)/(TEOSINTE BRANCHED 1) TB1 proteins. TCPs bind *cis*-acting regulatory elements (CAREs) known as site II, in the promoter regions of various genes. CAREs come in two classes: class I (GTGGGNCC) and class II (GTGGNCCC), which have different but similar binding preferences (Kosugi and Ohashi, 2002; Viola et al., 2012). The peculiar class I member TCP16 is an exception with preference for class II binding site since it contains Asp instead of Gly at a key discriminatory position (Uberti-Manassero et al., 2016).

A key mechanism underlying temporal control is the circadian clock. Among the central Arabidopsis clock components are the TFs CIRCADIAN CLOCK-ASSOCIATED 1 (CCA1) and LATE ELONGATED HYPOCOTYL (LHY) (for a review, see Nohales and Kay, 2016). Some TCP members interact with different components of the core circadian clock as shown in both yeast two-hybrid and protein–protein interaction assays (Giraud et al., 2010), which indicates that the TCP family is intricately linked to circadian regulation of gene expression in Arabidopsis. TCP21, termed CHE (for CCA1 Hiking Expedition), binds TOC1 (timing of CAB expression 1), which provides an explanation of how TOC1 can regulate expression of *CCA1*, as TOC1 lacks a DNA binding domain (Pruneda-Paz et al., 2009). Moreover, the concomitant binding of TCP20/TCP22 and LWD1 (LIGHT-REGULATED WD1) to the *CCA1* promoter activates its expression at dawn (Wu et al., 2016). Furthermore, TCPs appear to link the diurnal changes in mitochondrial function, particularly in genes encoding components of the oxidative phosphorylation machinery, with transcriptional changes that are regulated and integrated with the central clock function. This provides a molecular link between cellular and organelle metabolic activity and the circadian clock in plants (Palatnik et al., 2003; Welchen and Gonzalez, 2006; Giraud et al., 2010; Danisman, 2016).

Other developmental plant process that requires a TCP-mediated precise spatial and temporal control is the regulation of floral organ development, including secondary cell wall thickening necessary to release pollen grains. This developmental program is under the precise control of TCP24, which functions as a negative regulator (Wang et al., 2015). Moreover, the inhibition of the TCP16 function results in abortion of early pollen development (Takeda et al., 2006). In rice, class I TCP genes have been mainly implicated in stress adaptation, such as salinity tolerance (Almeida et al., 2017) or cold stress (Wang et al., 2014). OsTCP19 facilitates abiotic stress tolerance by manipulating the abscisic acid (ABA) signaling network (Mukhopadhyay and Tyagi, 2015). The wide crosstalk between TCP and hormones has been recently summarized (Nicolas and Cubas, 2016).

Environmental signals, such as nutrient availability, also lead to TCP-mediated regulation. In this sense, TCP20 and NIN-like proteins (NLP6 and NPL7) are involved in nitrate availability responses (Guan et al., 2014, 2017). This is only an example of the high range of TCPs interactions with other TFs (Bemer et al., 2017). These multiple interactions highlight the central role of TCPs in plant molecular networks that integrate environmental and endogenous processes in plants (Danisman, 2016; Dhaka et al., 2017). Metal availability is a key environmental factor under precise temporal control intricately linked with the circadian clock (Andrés-Colás et al., 2010; Hermans et al., 2010; Chen et al., 2013; Hong et al., 2013; Salomé et al., 2013). In this regard, it has been shown that TCP20 transcriptionally repress the expression of the subgroup Ib of bHLH TFs, previously implicated in iron homeostasis (Wang et al., 2007). Moreover, these TFs are up-regulated in the transition from cell proliferation to cell expansion during sink-source transitions (Andriankaja et al., 2014).

Although the activation of Cu^+^ uptake through the pmCOPT transporters under Cu deficiency by SLP7 is a well-established process (Yamasaki et al., 2009; Bernal et al., 2012), their temporal control, as well as the transcriptional regulation of imCOPTs, *COPT3* and *COPT5*, remain unsolved. In this work, we have identified TCP16 as a TF that, besides SPL7, could participate in Cu homeostasis via temporal modulation of gene expression in Arabidopsis.

## Materials and Methods

### Plant Growth Conditions and Treatments

*A. thaliana* plants, ecotype Col 0, and the transgenic lines indicated in Supplementary Table SI were grown as previously described (Andrés-Colás et al., 2010). The half-strength Murashige and Skoog (12 MS) medium was either commercial (Sigma) or prepared in the laboratory as follows: macronutrients 12.5 ml (NH_4_NO_3_ 825 mg/l, KNO_3_ 950 mg/l, MgSO_4_⋅7H_2_O 90.35 mg/l, KH_2_PO_4_ 85 mg/l, and CaCl_2_ 166.25 mg/l), micronutrients 0.5 ml (H_3_BO_3_ 3.1 mg/l, MnSO_4_⋅H_2_O 8.45 mg/l, ZnSO_4_⋅7H_2_O 4.3 mg/l, NaMoO_4_⋅2H_2_O 0.125 mg/l, and CoCl_2_⋅6H_2_O 0.0125 mg/l), Fe-EDTA 2.5 ml (FeSO_4_⋅7H_2_O 13.9 mg/l and Na_2_EDTA⋅2H_2_O 18.63 mg/l), KI 1.1 ml (0.41 mg/l), in both cases supplemented with MES 0.5 g/l, sucrose 10 g/l, agar 8 g/l, pH 5.7 with KOH. Variable CuSO_4_ concentrations were added when indicated. Media were supplemented with 100 μM BCS for the Cu-deprived media.

A *COPT3* promoter (*COPT3p*), covering 1,248 bp upstream from the start codon, was fused to the *uidA* (*GUS*) reporter gene (*COPT3p:GUS*) by substitution of the *CaMV35S* promoter in the pBI121 vector. At least two independent transgenic *Arabidopsis* stable homozygous lines harboring the *COPT3p:GUS* chimeric construct were obtained and analyzed.

The phenotype of two independent TRANSPLANTA (TPT) TPT TCP16 lines (Coego et al., 2014) was analyzed on 12 MS medium with 100 μM BCS, with or without 2 μM β-estradiol after germinating on 12 MS medium 2 days or for the indicated period, and under long (16 h light-23°C/8 h dark-16°C) or neutral (12 h light-23°C/12 h dark-16°C) photoperiod conditions, as indicated. For the gene expression analysis by RT-qPCR of TPT TCP16 lines, plants were grown directly with 2 μM β-estradiol. For this expression analysis, one TPT TCP16 line was used and compared to the wild-type. Samples were collected at 0, 12, or 24 h from plants grown under neutral conditions (0 or 24 h, start of light; 12 h, end of light), as indicated.

For the genotyping of the T-DNA insertion lines, plants were self-pollinated and homozygous lines were obtained. PCR (Supplementary Table SIV) or RT-qPCR (Supplementary Table SV) were performed with specific oligonucleotides to genotype or check the loss of expression in the lines, respectively. For the *tcp16* mutant, one line was analyzed and compared to the previously obtained TCP16 RNAi line. For the *copt3* mutant, one line was analyzed and compared to the COPT3-HA overexpressing line. For the *copt5* mutant, one of the previously characterized lines was used as a control for sensitivity to Cu deficiency.

### Electrophoretic Mobility Shift Assay (EMSA)

Biotin-labeled and unlabeled oligonucleotides (Supplementary Table SII), including the putative TCP binding motifs of *COPT3* and *COPT5* promoters, and a fragment of the *COPT2* promoter (without TCP binding motifs), were synthetized (VWR) and annealed in TEN buffer (10 mM Tris Base pH 7.8, 1 mM EDTA, 0.1 M NaCl) to generate the probes. The purified TCP16 and TCP23 proteins were obtained from the TRANSPLANTA consortium (Coego et al., 2014). Briefly, full-length *TCP16* and *TCP23* expression constructs were cloned in the destination vector pER8 and mobilized into pDONR201 using BP clonase reaction. cDNAs were transferred to destination vector pDEST-TH1 using LR clonase, yielding Maltose Binding Proteins (MBP) N-terminal fusions and constructs checked by sequencing. MBP-TCP16 and MBP-TCP23 constructs were transformed into BL-21 strain for expression. Induction of bacterial cultures was routinely at 25°C for 6 h with 1 mM Isopropyl β-D-1-thiogalactopyranoside. Expression of recombinant proteins was assessed by Western blot with an anti MBP antibody (BioLab) (Franco-Zorrilla et al., 2014). MBP-TCP16 and MBP-TCP23 proteins were bound to an amylose resin and eluted using maltose. EMSA was carried out with 960–1320 ng of purified protein, 0.01 pmol labeled probe and 225x unlabeled probe, as indicated, in binding buffer in 10 μl of total reaction volume. Protein buffer (2x TEN, 1 mM DTT, 1 mM protease inhibitor). Binding buffer (20 mM HEPES-KOH pH 7.8, 100 mM KCl, 1 mM EDTA, 0.1% BSA, 10 ng salmon sperm DNA, 10% glycerol). Binding reaction was performed at room temperature for 30 min. Electrophoresis was performed at 32 V on ice in a pre-run 5% native polyacrylamide gel in TBE buffer. Transfer was performed onto a nylon membrane at 40 V for 2 h on ice. Membrane crosslinking was at 120 mJ/cm^2^ 45–60 s at 254 nm in Stratalinker. Streptavidin-HRP conjugate antibody (Pr.Nr.21126, Pierce) was used for detection of the labeled probe. Relative bound DNA was quantified using ImageJ 1.42q software^1^. The experiment was repeated at least two independent times.

### Biochemical Fractionation

Plants overexpressing the COPT3-HA fusion protein (Andrés-Colás et al., 2010) were grown on soil. Chloroplasts were isolated and fractionated into stroma and thylakoids from leaves of 3- to 4-week-old plants as described (Pilon-Smits et al., 2002). Samples were normalized based on the number of chloroplasts and chlorophyll content, as determined by the method of Bruinsma (1961) as described (Pilon-Smits et al., 2002). Proteins were quantified by Bradford (1976) assay and separated by native 15% PAGE and then blotted on a nitrocellulose membrane. Immunodetection of SEC12, CpNifS, and PC was used as control of ER, stroma and thylakoids proteins with specific antiserum (Bar-Peled and Raikhel, 1997; Pilon-Smits et al., 2002; Abdel-Ghany et al., 2005). COPT3-HA was detected with anti-HA 3F10 specific antibody (Roche).

For sucrose density gradient fractionation the leaves of 4-week-old plants were ground with mortar and pestle in membrane isolation buffer [20 mM HEPES-KOH, pH 7; 50 mM C_2_H_3_KO_2_; 5 mM EDTA; 250 mM sorbitol, 1 mM DTT plus Complete^TM^ protease inhibitor cocktail (Roche)] and centrifuged at 2,000 × *g* for 10 min at 4°C. 3 ml of the supernatant were applied to the top of continuous 10 ml 20–60% (w/v) sucrose gradients, either with or without 5 mM MgCl_2_ added and centrifuged at 150,000 × *g* for 3 h at 4°C. Fractions of 0.5 ml were taken from the top and concentrated with TCA. The proteins in the fractions were electrophoresed in 12.5% SDS-PAGE, blotted and immunodetected using antibodies against the HA epitope (3F10, Roche), the ER SEC12, the plasma membrane α-AHA and the mitochondrial PMO35 markers, as described above.

### Subcellular Localization in *Arabidopsis* Protoplasts

The complete *COPT3* coding sequence was obtained from *Arabidopsis* genomic DNA by PCR using the following specific primers, which introduce the adequate restriction sites for cloning: C3-SalI F, 5′ CCACGCGTCGACATGAACGGCATGAGTGGATC; C3-NcoI R, 5′ CCATGCCATGGAACAATGTGATTGAACCTCGG. The C-terminus was fused with the GFP reporter and its expression was controlled by the constitutive CaMV35S promoter through its insertion into the transient expression vector pGFPau with the SpeI and SalI restriction enzymes.

The *COPT3-GFP* construct was used to transform *Arabidopsis* protoplasts obtained from the fresh leaf tissue of 3-week-old plants grown on soil, as previously described (Abdel-Ghany et al., 2005). After 16 h under continuous light at 23°C in the wash solution, confocal images were obtained using a fluorescence confocal microscope TCS SP vertical (DM-R) (Leica) equipped with an argon ion (458 and 488 nm), He-Ne I (543 nm) and He-Ne II (633 nm) excitation laser systems and a 60× objective lens. The fluorescence signals were detected at 500–530 nm for GFP and at 650–750 nm for chlorophyll, after exciting at 488 and 633 nm, respectively.

### GUS Staining and Pollen Preparations for Scanning Electron Microscopy

Assays were performed as described (Jefferson et al., 1987). Briefly, the organs were embedded with the substrate solution [100 mM NaPO_4_ pH 7.2, 0.5 mM K_3_Fe(CN)_6_, 0.5 mM K_4_Fe(CN)_6_, 0.1% (v/v) Triton X-100, 0.5 mM 5-bromo-4-chloro-3-indolyl-β-D-glucuronide (X-Gluc, AppliChem) and 10 mM EDTA pH 7.2]. Reactions took place at 37°C.

Pollen was mounted on standard stubs and coated with gold-palladium in a Bio-Rad E5600 ion sputter for 3 min prior to observation on a Hitachi S4100 FE scanning electron microscope. Digital images were acquired with the application EMIP.

### Cu Content Measurements

Cu content was determined by atomic absorption as described (Andrés-Colás et al., 2006; Carrió-Seguí et al., 2015) at the “*Servei Central de Suport a la Investigació Experimental SCSIE”* (Universitat de València) and the “*Servicios Centrales de Investigación*” (Universidad de Almería).

### Gene Expression by RT-PCR

Total RNA was isolated from *A. thaliana* seedlings with trizol reagent (Ambion). RNA was quantified by UV spectrophotometry and its integrity was visually assessed on ethidium bromide-stained agarose gels. Total RNA (1.5 μg) was first converted into cDNA by reverse transcription (RT) using SuperScript II reverse transcriptase (Invitrogen) and anchored oligo(dT)_15_ (Roche) and 18S reverse primer. PCR was performed under the following conditions to maintain a linear response in the range of the cDNA concentrations used (see Supplementary Table SIV for the primer-specific sequences): 30 cycles, except 20 cycles for *18S*, of three temperature segments of 30 s (Td 94°C/Th 55°C/Te 72°C). The PCR products were visualized in 2% agarose gels. Real-time PCRs (qPCR) were carried out with *SYBR-Green qPCR Super-Mix-UDG* with *ROX* (Invitrogen) and specific oligonucleotides (Supplementary Table SV) in a *StepOnePlus Real-Time PCR System* (Applied Biosystems) under 1 cycle of 95°C for 2 min and 40 cycles consisting in 95°C for 30 s and 60°C for 30 s. The results correspond to the comparative Ct (cycle threshold) method (ΔΔCt). The *UBQ10* gene was used as a loading control. Values are relative expression with respect to the first sample in each graph, in arbitrary units.

### Computer-Assisted Sequence

The theoretical promoter sequences analysis was performed by Patmatch from TAIR ^2^.

## Results

### COPT3 Protein Was Intracellular Localized

The *COPT3* gene (At5g59040) is located on chromosome V of the *A. thaliana* genome, adjacent to *COPT1.* The two genes are organized head-to-head in opposite orientations separated by 2,266 bp (Supplementary Figure S1A). The *COPT3* gene encodes a 151 amino acid protein that displays features, which are conserved in COPT/CTR-type transport proteins. These conserved features include three transmembrane domains (TMDs) with an external amino terminus, containing a conserved Met residue and a cytosolic carboxy terminus, as well as the Mx_3_M and the Gx_3_G motifs within TMD2 and TMD3, respectively (Peñarrubia et al., 2010; Puig, 2014).

The 1.4 kb promoter region of the *COPT3* gene contains a number of potential *cis* regulatory elements (Supplementary Figure S1B). One of these is a putative plastid expression box at position -388. Sequence analysis of the coding region suggested that a putative transit sequence for targeting to the chloroplast may be present in COPT3 (*PSORT*^3^) (Supplementary Figure S1C). In order to analyze its subcellular localization, chloroplasts were isolated from an *Arabidopsis* transgenic line expressing the *COPT3* coding sequence tagged with the human influenza hemagglutinin (HA) epitope under the control of the *35S* cauliflower mosaic virus (*CaMV35S*) promoter (*COPT3-HA*) (Andrés-Colás et al., 2010). The analysis of isolated chloroplast fractions clearly indicated that COPT3 was not present in plastids (Supplementary Figures S2A,C).

**FIGURE 1:**
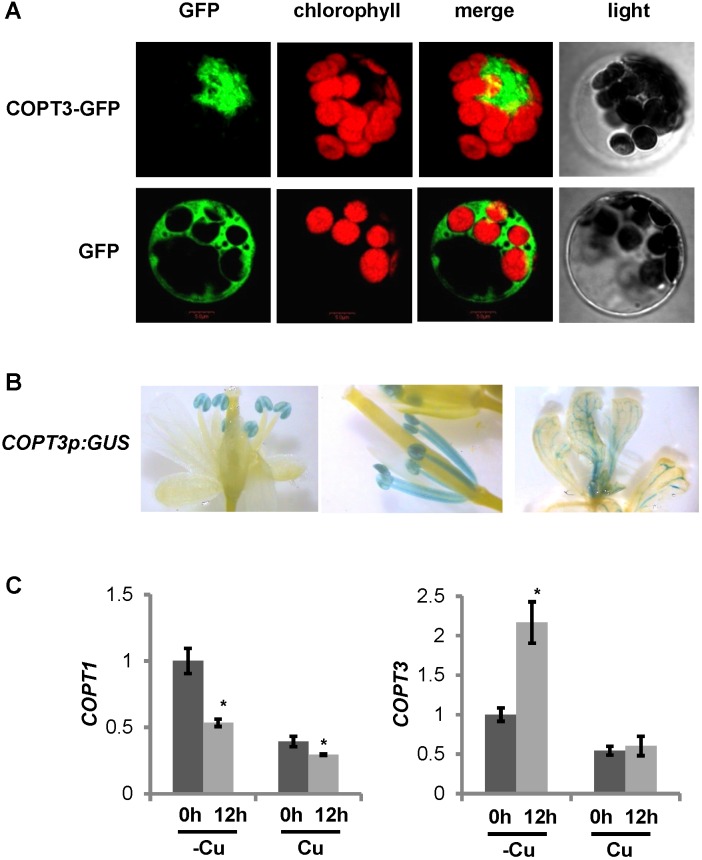
COPT3 expression pattern and regulation by Cu. **(A)** COPT3 subcellular localization. *Arabidopsis* protoplasts isolated from 30-day-old leaves and transiently transformed with the *35S:COPT3-GFP* construct or the *35S:GFP* as a control, were analyzed by confocal microscopy at 16 h post transformation. Green and red fluorescence are indicative of the localization of the GFP protein and the chlorophyll, respectively. Representative protoplasts from at least two independent experiments are shown on the same scale, including their merged and light fields. **(B)** Histochemical β-glucuronidase staining of the *Arabidopsis* lines transformed with the *COPT3p:GUS* construct. A representative flower (left), mature anthers and filaments (middle) and seedling shoot (right) from at least two independent lines are shown. **(C)** *COPT1* and *COPT3* temporal expression pattern and regulation by Cu. Seven-day-old wild-type seedlings grown under Cu deficiency (12 MS with 100 μM BCS; –Cu) and Cu sufficiency conditions (12 MS with 1 μM CuSO_4_; Cu). Samples were taken at 0 h (dark bars) and 12 h (light bars) of the 12 h light/12 h dark cycle. Total RNA was extracted and analyzed by RT-qPCR with specific oligonucleotides for *COPT1* and *COPT3*. The relative expression in arbitrary units is represented. Values correspond to arithmetic means (2^-ΔΔCt^) ± standard deviation from three biological replicates with three technical replicates for each (*n* = 3). Asterisks indicate significant differences for the same group of samples with respect to the 0 h sample (*P* < 0.05) according to *T*-test.

Next, COPT3 subcellular localization was analyzed by transient expression in *Arabidopsis* protoplasts of the *COPT3* coding region tagged with the green fluorescence protein (GFP) under the control of the *CaMV35S* promoter (*COPT3-GFP*) (Supplementary Table SI). The signal obtained confirmed an intracellular localization of COPT3 excluding the plasma membrane and chloroplasts (**Figure 1A**). Moreover, sucrose density gradient fractionation of membranes from leaves of plants expressing the *COPT3-HA* construct indicated that the COPT3 distribution pattern is more similar to the ER protein marker SEC12 than to the other markers, such as the mitochondrial PMO35 protein or the plasma membrane α-AHA protein (Supplementary Figures S2B,D). These results point to a putative COPT3 localization in the endomembrane system, maybe in the ER.

### *COPT3* Was Mainly Expressed in Pollen and Vascular Bundles

The analysis of the *COPT3* promoter (Supplementary Figure S1B) indicated the presence of several GTGA boxes (7), described in the late pollen *g10* gene promoter (Rogers et al., 2001). Additionally, one of the two co-dependent elements responsible for the pollen-specific activation of tomato *LAT52* gene (Bate and Twell, 1998), the AGAAA element, were present at multiple positions (12). There were also several boxes for expression in embryo (3), endosperm (4) and aleurone (2) (Supplementary Figure S1B).

In order to determine the tissue expression pattern of *COPT3* throughout the plant, we first performed RT-PCR analysis from various organs of adult plants. The result indicated that *COPT3* expression was detected in flowers and dried seeds, but also in stems and leaves, whereas it was hardly detectable in roots (Supplementary Figure S3A). These results were consistent with the Genevestigator database^4^, which also indicated that overall levels of *COPT3* expression were low throughout the plant life cycle compared to other COPT family members.

To further study the *COPT3* spatial expression pattern, stable transgenic *Arabidopsis* lines harboring the *GUS* reporter gene driven by the *COPT3* promoter (*COPT3p:GUS*) were obtained (Supplementary Table SI). During the development of reproductive organs, GUS staining was observed in anthers with a strong signal in pollen (**Figure 1B**, left). Accordingly, GUS staining was detected in the stamen filaments only when the styles were elongating (**Figure 1B**, middle). Moreover, GUS staining was also detected in the leaf vascular bundles (**Figure 1B**, right).

### COPT3 Expression Was Up-Regulated at Dusk and Down-Regulated by Cu

The *COPT3* promoter also displayed regulatory elements conserved in light regulated genes, denoted as I-box (GATAA), as well as an element (CAANNNNATC) required for the tomato *LHC* circadian expression and an Evening Element (AAAATATCT) involved in circadian regulation (Terzaghi and Cashmore, 1995; Harmer et al., 2000; Rawat et al., 2005) (Supplementary Figure S1B). Moreover, based on the DIURNAL DataBase^5^ (Mockler et al., 2007), *COPT3* expression oscillated with a phase of around 24 h under different circadian and diurnal conditions and peaked at 12 h (end of light period) of the 12 h light/12 h dark neutral photoperiod cycle (Supplementary Figure S3B). Furthermore, it was previously shown that altered Cu transport, through *COPT1* and *COPT3* overexpression, affected circadian rhythms regulation (Andrés-Colás et al., 2010). Taken together, these data may indicate a *COPT3* temporal regulation. As a first approach to address its study, *COPT3* expression was tested at 0 and 12 h in 7-day-old seedlings grown on neutral photoperiod conditions. The result confirmed the higher *COPT3* expression at 12 h (end of the light period), opposite to *COPT1* expression that was higher at 0 h (start of the light period), under Cu deficiency (**Figure 1C**). This result confirmed our previous data on the temporal expression of other *pmCOPT* members, such as *COPT2* and *COPT6,* both peaking at dawn (Perea-García et al., 2016a), whereas the other *imCOPT*, *COPT5,* peaked at dusk (not shown). These data are also in agreement with data in the DIURNAL DataBase for the *pmCOPT* and *imCOPT* expression, where both subfamily member types oppositely oscillated during the diurnal cycle.

Moreover, the *COPT3* promoter displayed three putative Cu deficiency response elements (GTAC) (Supplementary Figure S1B), previously described in the promoters of Cu-deficiency regulated genes that may be target sites for SPL7 (Yamasaki et al., 2009; Bernal et al., 2012). Although *COPT3* expression was previously reported to be independent of the Cu levels (Sancenón et al., 2003), the presence of these elements suggested a possible up-regulation of *COPT3* under Cu deficiency. In order to check if *COPT3* was differentially regulated by Cu over day and night, samples of seedlings grown under Cu deficiency and excess were checked by RT-qPCR at 0 and 12 h (**Figure 1C**). *COPT3* expression was significantly increased under Cu deficiency, specifically at 12 h (end of the light period). Furthermore, a wide range of Cu concentrations were tested and, in general, higher *COPT3* expression levels were observed in Cu deficiency media when compared to Cu sufficiency or Cu excess (Supplementary Figure S3C). However, the temporal specific increase of *COPT3* expression under Cu deficiency at 12 h, did not correlate to the expression peak of SPL7 at 0 h (Perea-García et al., 2016a).

### Two Independent TPT TCP16 Lines Were Sensitive to Cu Deficiency

In order to find regulatory factors involved in the temporal pattern of *COPT3* expression, a screen of a conditional overexpression TF library, denoted TRANSPLANTA (TPT) (Coego et al., 2014), was performed under Cu deficiency conditions. The TRANSPLANTA collection contains 634 *Arabidopsis* TFs transferred into a vector (pER8) that conferred a β-estradiol-inducible gene overexpression (Zuo et al., 2000). At least, two independent single insertion and homozygous transgenic lines were generated for each TF (Coego et al., 2014). Reporter lines under the same promoter (*pER8G:GUS-GFP*) were used to optimize the expression conditions in our experimental set up (Supplementary Figure S4A). The treatment with 2 μM β-estradiol induced reporter expression after 12 h and treatment with 100 μM BCS did not modify *GUS* expression (Supplementary Figure S4B). Based on these results, a screen was performed in 7-day-old seedlings germinated on 12 MS medium and then grown on 100 μM BCS with 2 μM β-estradiol. Afterward, seedlings were transferred to fresh plates containing 10 μM Cu and 2 μM β-estradiol for checking the recovery of root growth with the aim to discard lines with phenotypes unrelated to Cu deficiency. The *copt5* mutant line, which exhibited a defect in root elongation under Cu deficiency (Garcia-Molina et al., 2011) and WT seedlings were used as controls (Supplementary Figure S4C).

One of the TF families in the TPT lines with a higher percentage of the members showing a short root phenotype in the screen under Cu deficiency, which was reverted under high Cu, was the TCP family (not shown). The TRANSPLANTA collection contains TPT lines for 17 TCPs out of a total of 24 members present in the *Arabidopsis* genome. TPT lines from six of them (TCP14, TCP16, TCP19, TCP20, TCP22, and TCP24) displayed a short root phenotype under Cu deficiency, which reverted under high Cu (**Table 1**). All of them belong to the TCP class I PCF, except TCP24. It is noteworthy that most TCP factors display putative Cu deficiency responsive GTAC boxes in their proximal promoters (500 bp), except TCP4 and TCP17 (**Table 1**). Thus, TPT lines positive in the screening corresponded to TCPs that all contain GTAC boxes. Among them, TCP16 (At3g45150) was chosen for further study since the similar curly leaves phenotype found in plants where TCP16 was fused to a repressor domain (Uberti-Manassero et al., 2016) and in plants overexpressing *COPT1* and *COPT3* (Andrés-Colás et al., 2010; Garcia-Molina et al., 2013).

**Table 1:** TCPs characteristics and screening results.

TCP	MIPS code	Class	Type	Transplanta	Screening	Class I CAREs GGNCCCAC TGGGCC GCCCR GG(A/T)CCC	Class II CAREs G(T/C)GGNCCC GGACCA	Other CARE versions	CuRE
TCP1	At1g67260	II	CYC/TB1	+	-			ATGGATCCAA	4^∗^
TCP2	At4g18390	II	CIN	+	-	+		0	1
TCP3	At1g53230	II	CIN	-	N.D.			0	1
TCP4	At3g15030	II	CIN	-	N.D.	+		0	0
TCP5	At5g60970	II	CIN	+	-			0	3
TCP6	At5g41030	I	PCF	+	-			0	3
TCP7	At5g23280	I	PCF	-	N.D.	+	+	GTGAGCTCCA	2
TCP8	At1g58100	I	PCF	+	N.D.			0	1
TCP9	At2g45680	I	PCF	+	-	+		ATGGTCCCAT	5^∗^
TCP10	At2g31070	II	CIN	-	N.D.			GTGGGCAACA	1
TCP11	At2g37000	I	PCF	+	-	+		0	5
TCP12	At1g68800	II	CYC/TB1	-	N.D.			0	4
TCP13	At3g02150	II	CIN	-	N.D.			0	3^∗^
TCP14	At3g47620	I	PCF	+	+			0	1
TCP15	At1g69690	I	PCF	+	-	+		0	4
TCP16	At3g45150	I	PCF	+	+			GTGGACCTAT TCAGGTCCAC	2
TCP17	At5g08070	II	CIN	+	-			0	0
TCP18	At3g18550	II	CYC/TB1	+	-			0	3
TCP19	At5g51910	I	PCF	+	+	+		GTGGTCGAGG	2
TCP20	At3g27010	I	PCF	+	+			0	1
TCP21	At5g08330	I	PCF	-	N.D.	+	+	GTGGTCCAAC	2
TCP22	At1g72010	I	PCF	+	+			0	3
TCP23	At1g35560	I	PCF	+	-			GTTAGACCAA TTCGGCGCAT GTGGAAACAG GTGGGACTAC	2
TCP24	At1g30210	II	CIN	+	+			0	3

*TRANSPLANTA, available TPT lines (+) or not (-). Screening, germinated in 12 MS medium, grown in 100 μM BCS and recovered with 10 μM Cu after 100 μM BCS. Class I/II CAREs, presence (+) of class I/II TCP CAREs as indicated. Other CARE versions, presence of different versions of the TCP motifs as indicated. CuRE, number of GTAC motifs in the first 500 bp upstream (^∗^, 3 GTAC motifs along 65 bp). Motifs were considered in the first 500 bp upstream and in both strains.*

**FIGURE 2:**
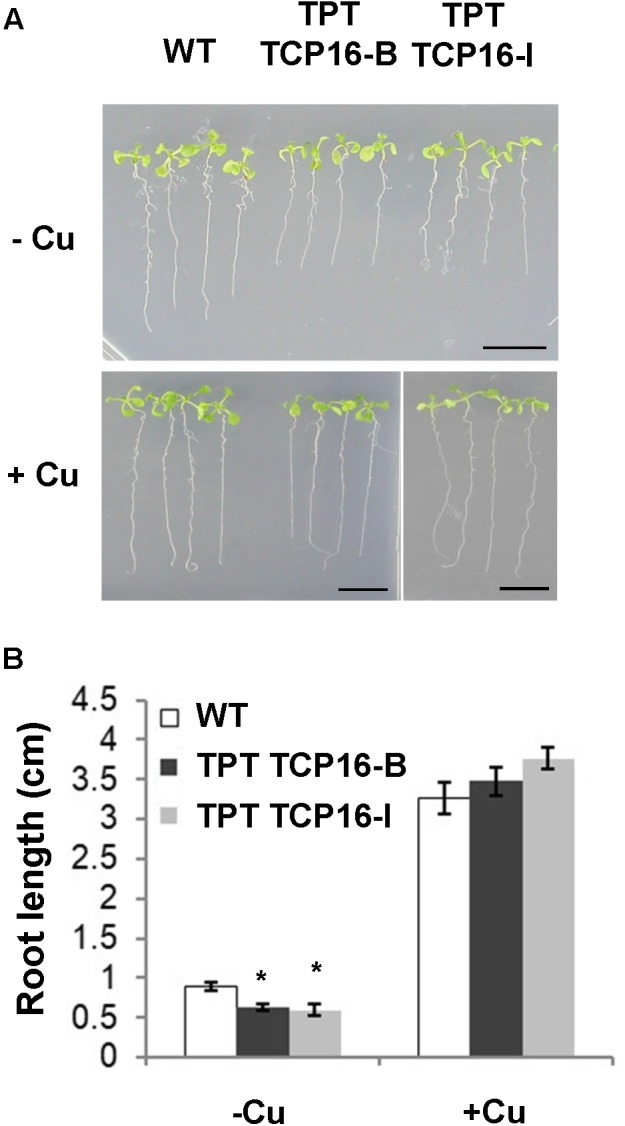
Effect of Cu on root length in the TCP16 TPT lines. **(A)** Photograph of representative 8-day-old wild-type (WT), TCP16-B and TCP16-I TPT seedlings grown in 12 MS medium supplemented with 100 μM BCS (–Cu) or 10 μM CuSO_4_ (+Cu), in the presence of 2 μM β-estradiol, under 12 h light/12 h dark cycles. A representative image of at least three independent experiments is shown. Scale bars, 1 cm. **(B)** Root length of 7-day-old WT and TCP16-B and TCP16-I TPT seedlings grown under the same conditions as indicated in **(A)**. Bars correspond to means ± standard deviation of three biological replicates of at least 15 plants each. Asterisks indicate statistical differences (*P* < 0.05) with respect to the WT value, according to Tukey test.

Two independent TPT TCP16 lines, TPT 3.45150.1B (TPT TCP16-B) and TPT 3.45150.1I (TPT TCP16-I) (Supplementary Table SI), were sensitive to Cu deficiency, as shown by a reduced root elongation (**Figure 2A**). The reduced root length was specifically linked to the Cu deficiency since the root length defect was not observed in the presence of excess Cu (**Figure 2**). Moreover, this phenotype was indeed due to the induced overexpression of *TCP16* since it was not observed in the TPT TCP16 lines in the absence of β-estradiol (Supplementary Figure S4C).

### The TCP16 Transcription Factor Bound the *COPT3* Promoter

The promoters of genes participating in Cu homeostasis were analyzed for the presence of putative *cis* CARE elements recognized by TCPs. This indicated that *COPT3* and *COPT5* were the only members of the COPT family that displayed putative CAREs (Supplementary Table SII). These CARE elements in the *COPT3* (TTGAGCCCAT) and *COPT5* (GTGAGCCCAC) promoters where identified as a specific version of the previously described TCP16 CARE (Martín-Trillo and Cubas, 2010).

**FIGURE 3:**
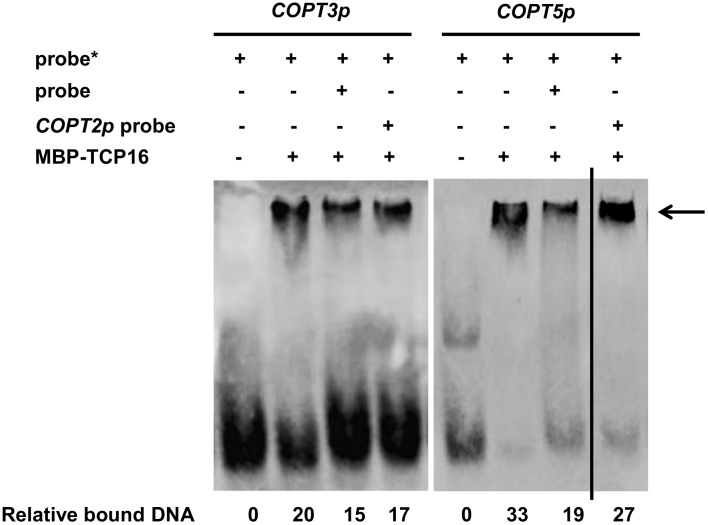
EMSA binding assays between TCP16 protein and *COPTs* promoters. Immunoblot of purified TCP16 protein incubated with biotin-labeled (^∗^) DNA probe from *COPT3* and *COPT5* promoters, containing TCP box. Unlabeled probes were added as a competitor as indicated. The arrow indicates the bound DNA. Relative intensity of the bands corresponding to bound DNA is shown. Representative images of at least two independent experiments are shown.

In order to check if TCP16 had a direct effect on *imCOPTs* regulation, binding to the promoter regions containing the putative CARE elements in *COPT3* and *COPT5* promoters (Supplementary Table SIII) was analyzed by Electrophoretic Mobility Shift Assay (EMSA) with the purified TCP16 protein (provided by the TRANSPLANTA consortium) (Coego et al., 2014). TCP16 interacted with the *COPT3* promoter, as shown by a retarded *COPT3* probe band in the presence of the TCP16 protein (**Figure 3**). An excess of *COPT3* unlabeled probe reduced the TCP16 binding, as shown by a lower intensity of the retarded *COPT3* probe band in the presence of the TCP16 protein. However, with the same amount of the *COPT2* unlabeled probe (the *COPT2* promoter has no CARE; Supplementary Table SIII), a minor reduction was observed, pointing to the specificity of the TCP16 interaction with the *COPT3* promoter. As well as *COPT3*, TCP16 also bound the *COPT5* promoter and specifically compete with an excess of the *COPT5* but not of the *COPT2* unlabeled probe (**Figure 3**). TCP23 is a class I TCP member involved in plant development (Balsemão-Pires et al., 2013) that resulted negative in the TPT screening (**Table 1**). Under the same experimental conditions, no interactions of TCP23 with the *COPT3* (Supplementary Figure S5A) and the *COPT5* promoters (Supplementary Figure S5B) were detected. Taken together, the TCP16 TF specifically bound to the *COPT3* and *COPT5* promoters *in vitro*.

### TCP16 Was Involved in Repression of *COPT3* Expression

**FIGURE 4:**
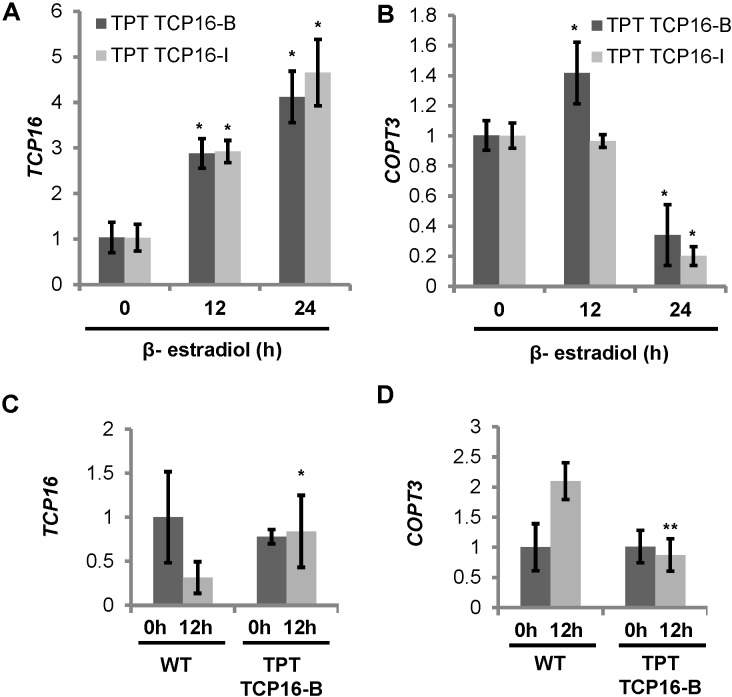
Expression of *TCP16* and *COPT3* under different conditions in the TCP16 TPT lines. **(A,B)** Kinetics of *TCP16* and *COPT3* expression in TCP16 TPT lines by β-estradiol. Seven-day-old TCP16-B (dark bars) and TCP16-I (light bars) TPT seedlings grown in 12 MS medium supplemented with 100 μM BCS. Samples were taken at indicated times, after treatment with 2 μM β-estradiol at 0 h of the 12 h light/12 h dark cycle in day 7. Total RNA was extracted and analyzed by RT-qPCR with specific oligonucleotides for *TCP16* **(A)** and *COPT3* **(B)**. The relative expression in arbitrary units is represented. Values correspond to arithmetic means (2^-ΔΔCt^) ± standard deviation from three biological replicates with three technical replicates for each (*n* = 3). Asterisks indicate significant differences (*P* ≤ 0.05) according to *T*-test. **(C,D)** *TCP16* and *COPT3* temporal expression. Six-day-old wild-type (WT) and TPT TCP16-B seedlings grown in 12 MS medium supplemented with 100 μM BCS and 2 μM β-estradiol. Samples were taken at 0 h (dark bars) and 12 h (light bars) of the 12 h light/12 h dark cycle. Total RNA was extracted and analyzed by RT-qPCR with specific oligonucleotides for *TCP16* **(C)** and *COPT3* **(D)**. The relative expression in arbitrary units is represented. Values correspond to arithmetic means (2^-ΔΔCt^) ± standard deviation from at least three biological replicates (*n* ≥ 3). Asterisks indicate significant differences for the same group of samples respect to the WT line (^∗^*P* ≤ 0.1; ^∗∗^*P* ≤ 0.04) according to *T*-test.

To check the effect of TCP16 on *COPT3* expression, *TCP16* and *COPT3* mRNA levels were determined in 7-day-old TPT TCP16 seedlings at different times after β-estradiol induction. Short-term kinetics indicated that *TCP16* expression levels increased 4–5 times after 24 h induction in both lines (**Figure 4A**). In parallel to the increase in *TCP16* expression at 24 h after induction, *COPT3* expression levels were reduced to 25–15% (**Figure 4B**). Besides, long-term *TCP16* overexpression by β-estradiol was dependent on the diurnal time, being higher at 12 h of the neutral photoperiod cycle (**Figure 4C**) and provoked a repression of *COPT3* expression, specifically at this time of the day, under Cu deficiency (**Figure 4D**). As a control, the *GFP-GUS* line showed similar levels of *GUS* overexpression at 0 and 12 h of the neutral photoperiod cycle (Supplementary Figure S4A) and other TPT lines showed a robust β-estradiol-dependent induction of the TF transgene although to a different extent depending on the line (Coego et al., 2014). These data pointed to a repressive TCP16 role on *COPT3* expression.

### TCP16 Expression Was Up-Regulated at Dawn and by Cu

**FIGURE 5:**
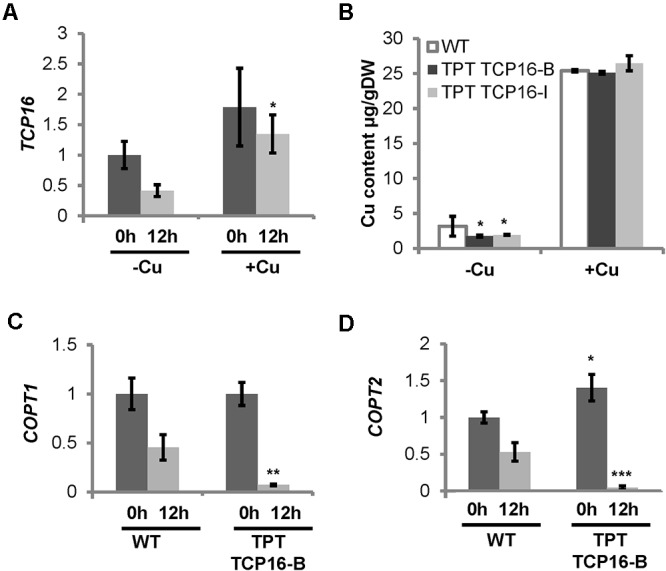
*TCP16* temporal expression pattern, regulation by Cu, and Cu status in the TCP16 TPT lines. **(A)** *TCP16* expression. Six-day-old wild-type seedlings grown in 12 MS medium (MS) and the same medium supplemented with 100 μM BCS (–Cu) or 10 μM CuSO_4_ (+Cu). Samples were taken at 0 h (dark bars) and 12 h (light bars) of the 12 h light/12 h dark cycle. Total RNA was extracted and analyzed by RT-qPCR with specific oligonucleotides for *TCP16*. The relative expression in arbitrary units is represented. Values correspond to arithmetic means (2^-ΔΔCt^) ± standard deviation from at least three biological replicates (*n* ≥ 3). Asterisk indicates significant differences for the same group of samples with respect to the other conditions (*P* ≤ 0.002) according to *T*-test. **(B)** Cu content in TPT TCP16 plants. Seven-day-old wild-type (white bars), TCP16-B (dark gray bars) and TCP16-I (light gray bars) TPT seedlings grown in 12 MS medium supplemented with 100 μM BCS (–Cu) or with 10 μM CuSO_4_ (+Cu), in the presence of 2 μM β-estradiol, under 12 h light/12 h dark cycles. Values correspond to arithmetic means ± standard deviation from three biological replicates of 120 mg of DW. Asterisks indicate significant differences between WT and mutant lines in the same condition (*P* ≤ 0.05) according to *T*-test. **(C,D)** Cu-status marker expression in TPT TCP16 line. Six-day-old wild-type (WT) and TPT TCP16-B seedlings grown in 12 MS medium supplemented with 100 μM BCS and 2 μM β-estradiol. Samples were taken at 0 h (dark bars) and 12 h (light bars) of the 12 h light/12 h dark cycle. Total RNA was extracted and analyzed by RT-qPCR with specific oligonucleotides for *COPT1* **(C)** and *COPT2* **(D)**. The relative expression in arbitrary units is represented. Values correspond to arithmetic means (2^-ΔΔCt^) ± standard deviation from four biological replicates (*n* = 4). Asterisks indicate significant differences for the same group of samples with respect to the WT line (^∗^*P* ≤ 0.02; ^∗∗^*P* ≤ 0.005; ^∗∗∗^*P* ≤ 0.0005) according to *T*-test.

Since the *TCP16* promoter contains a putative SPL7-responsive GTAC box (**Table 1**), we checked its expression in WT seedlings in the same samples that were used for *COPT3* expression analysis (**Figure 1C**). The results indicated that *TCP16* expression was higher at 0 h than at 12 h of the 12 h light/12 h dark cycle, under Cu deficiency (**Figure 5A**). These results may point to a diurnal oscillation of *TCP16* expression opposite to the one shown by *COPT3*. Unfortunately, the *TCP16* expression pattern is unavailable in the DIURNAL DataBase to be compared with the results shown here. Moreover, *TCP16* expression was significantly higher under Cu excess at 12 h with respect to Cu deficiency, thus high transcript levels remained along day and night under Cu excess (**Figure 5A**), coinciding with the reduction in *COPT3* expression (**Figure 1C**). Taken together, these data pointed to a role for TCP16 as a temporal transcriptional repressor of *COPT3*, specially at dawn under Cu deficiency and along day and night under Cu excess.

Cu levels were determined in the conditionally overexpressing TPT TCP16-B and TPT TCP16-I lines (**Figure 5B**). TPT TCP16 lines had lower Cu content than WT under Cu deficiency. This result pointed to affected Cu uptake. To test this possibility, the expression of *pmCOPTs* (*COPT1* and *COPT2*) was analyzed. Both *pmCOPT* were selectively reduced at 12 h in TPT TCP16-B under Cu deficiency conditions (**Figures 5C,D**), despite the absence of TCP16 binding boxes in their promoters (Supplementary Table SII).

### The Loss-of-Function of TCP16 Exhibited Copper-Related Phenotypes

**FIGURE 6:**
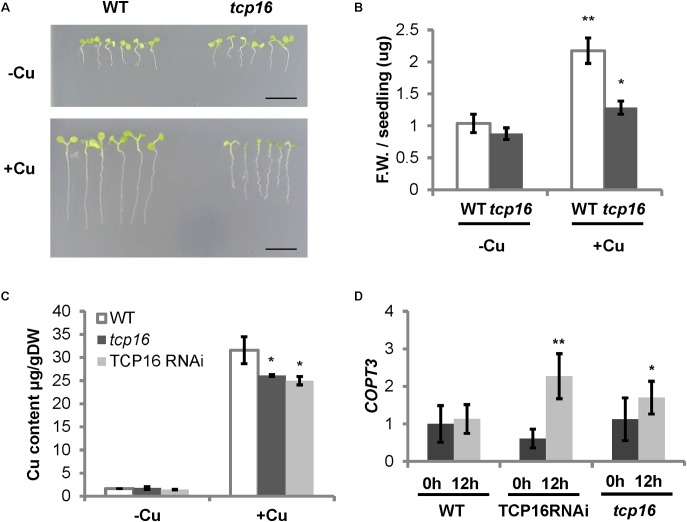
Phenotype of *tcp16* seedlings under different Cu status and *COPT3* expression in TCP16 RNAi and knockout lines. **(A)** Photographs of representative 6-day-old wild-type (WT) and *tcp16* seedlings grown in 12 MS medium supplemented with 100 μM BCS (–Cu) or 10 μM CuSO_4_ (+Cu) under 12 h light/12 h dark cycles. A representative image of at least three independent experiments is shown. Scale bars, 1 cm. **(B)** Fresh weight of wild-type (white bars) and *tcp16* (dark gray bars) 7-day-old seedlings grown in the same conditions shown in **(A)**. Bars correspond to means ± standard deviation of six biological replicates of five seedlings each. Asterisks indicate significant differences (^∗^*P* < 0.05; ^∗∗^*P* < 0.01) according to Duncan test. **(C)** Cu content in WT (white bars), TCP16 RNAi (dark gray bars) and *tcp16* (light gray bars) 7-day-old seedlings grown in the same conditions shown in **(A)**. Bars correspond to means ± standard deviation of three biological replicates of 120 mg of DW. Asterisks indicate statistical differences between WT and mutant lines in the same condition (*P* < 0.05) according to *T*-test. **(D)** *COPT3* expression. Six-day-old wild-type (WT), TCP16 RNAi and *tcp16* seedlings grown in 12 MS medium supplemented with 10 μM CuSO_4_. Samples were taken at 0 h (dark bars) and 12 h (light bars) of the 12 h light/12 h dark cycle. Total RNA was extracted and analyzed by RT-qPCR with specific oligonucleotides for *COPT3*. The relative expression in arbitrary units is represented. Values correspond to arithmetic means (2^-ΔΔCt^) ± standard deviation from at least three biological replicates (*n* ≥ 3). Asterisks indicate significant differences for the same group of samples with respect to the WT line (^∗^*P* ≤ 0.06; ^∗∗^*P* ≤ 0.009) according to *T*-test.

In order to have a better understanding of the role of TCP16 in regulating Cu homeostasis, we used both a RNA interference line TCP16RNAi (Takeda et al., 2006) and a T-DNA insertion mutant *tcp16* (N462818) (Supplementary Table SI). The *tcp16* mutant contains the T-DNA insert at +110 bp of the *TCP16* coding sequence (Supplementary Figures S6A,B). A homozygous *tcp16* line was selected (Supplementary Figure S6C) and the loss of the *TCP16* expression at 0 h corroborated by RT-qPCR (Supplementary Figure S6D). The growth of *tcp16* was checked under Cu deficiency and excess in the medium. The *tcp16* seedlings showed defects in root elongation and fresh weight mostly under Cu excess (**Figures 6A,B**), coincident with the conditions where *TCP16* was mainly expressed (**Figure 5A**). Moreover, Cu content was also determined by atomic absorbance and, whereas conditionally overexpressing TPT TCP16 lines had lower Cu content than WT under Cu deficiency (**Figure 5B**), a decreased level of Cu content was observed in the loss-of-function *TCP16RNAi* and *tcp16* lines under Cu excess (**Figure 6C**).

To check the effect of the TCP16 loss-of-function on *COPT3* expression, *COPT3* mRNA levels were determined in 7-day-old seedlings of the *TCP16RNAi* and *tcp16* lines under Cu excess. An increase in *COPT3* expression levels was observed specifically at the end of the light period (12 h) (**Figure 6D**). This effect was the opposite to the one observed in the conditionally overexpressing TPT TCP16-B line (**Figure 4D**), further pointing to the function of TCP16 as a repressor of *COPT3*.

### COPT3 Function Was Required for Proper Repression of *TCP16*

**FIGURE 7:**
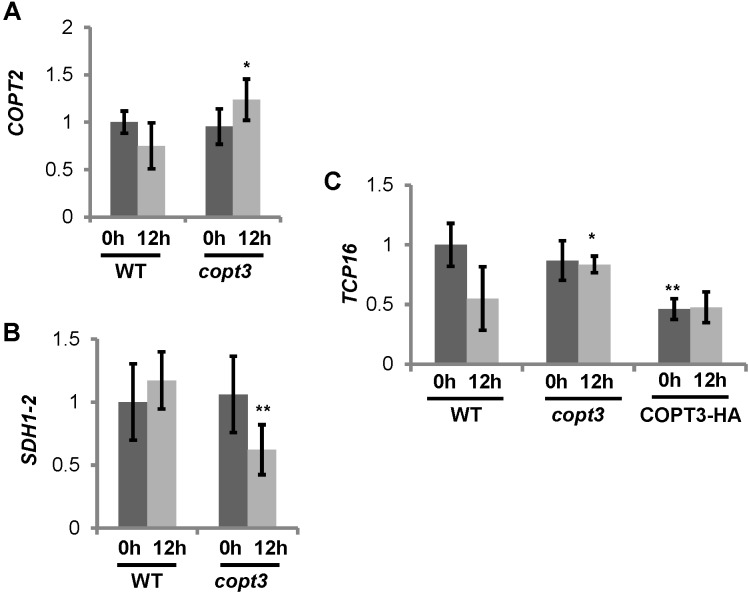
Phenotype of *copt3* knockout seedlings under different Cu status. **(A,B)** Cu-status marker expression in *copt3* lines. Flowers from wild type (WT) and *copt3* knockout plants grown in soil. Samples were taken at 0 h (dark bars) and 12 h (light bars) of the 12 h light/12 h dark cycle. Total RNA was extracted and analyzed by RT-qPCR with specific oligonucleotides for *COPT2* (A) and *SDH1-2* **(B)**. The relative expression in arbitrary units is represented. Values correspond to arithmetic means (2^-ΔΔCt^) ± standard deviation from at least three biological replicates (*n* ≥ 3). Asterisk indicates significant differences for the same group of samples with respect to the WT line (^∗^*P* ≤ 0.04; ^∗∗^*P* ≤ 0.03) according to *T*-test. **(C)** *TCP16* expression in *copt3* and COPT3^OE^ lines. Six-day-old wild-type (WT), *copt3* and COPT3^OE^ seedlings grown in 12 MS medium supplemented with 100 μM BCS. Samples were taken at 0 h (dark bars) and 12 h (light bars) of the 12 h light/12 h dark cycle. Total RNA was extracted and analyzed by RT-qPCR with specific oligonucleotides for *TCP16*. The relative expression in arbitrary units is represented. Values correspond to arithmetic means (2^-ΔΔCt^) ± standard deviation from at least two biological replicates (*n* ≥ 2). Asterisks indicate significant differences for the same group of samples with respect to the WT line (^∗^*P* ≤ 0.15; ^∗∗^*P* ≤ 0.005) according to *T*-test.

The possibility that COPT3 function could reciprocally affect *TCP16* expression was also checked. For that purpose, we analyzed the *TCP16* expression in transgenic lines with altered *COPT3* expression levels and used the *COPT3-HA* line (Andrés-Colás et al., 2010) and loss-of-function *copt3* mutant (GK633G06) (Supplementary Table SI). The *copt3* mutant contains a T-DNA insertion at +109 bp of the *COPT3* coding sequence (Supplementary Figure S7A). A homozygous *copt3* line was selected (Supplementary Figure S7B) and the loss of the *COPT3* expression in flowers corroborated by RT-qPCR (Supplementary Figure S7C). The COPT3-HA line was sensitive to Cu excess, as shown by a reduction in root elongation and alteration of the flower morphology and flowering time (Andrés-Colás et al., 2010). On the contrary, the *copt3* mutant was more sensitive to Cu deficiency than WT and accordingly accumulated less Cu than controls (not shown). The levels of the Cu deficiency marker *COPT2* were higher at 12 h in the *copt3* mutant (**Figure 7A**), whereas *SDH1-2*, a mitochondrial marker of Cu excess (Andrés-Colás et al., 2013), was significantly reduced at this time in flowers (**Figure 7B**) further pointing to decreased Cu levels in the *copt3* mutant.

The expression analysis showed that *TCP16* levels were higher at 12 h in the *copt3* mutant line, in contrast with a lower expression at 0 h in the *COPT3-HA* transgenic line (**Figure 7C**). These data suggested that, in addition to the already mentioned repression of TCP16 over *COPT3*, a reciprocal repressing effect of the COPT3 function on *TCP16* expression was taking also place.

**FIGURE 8:**
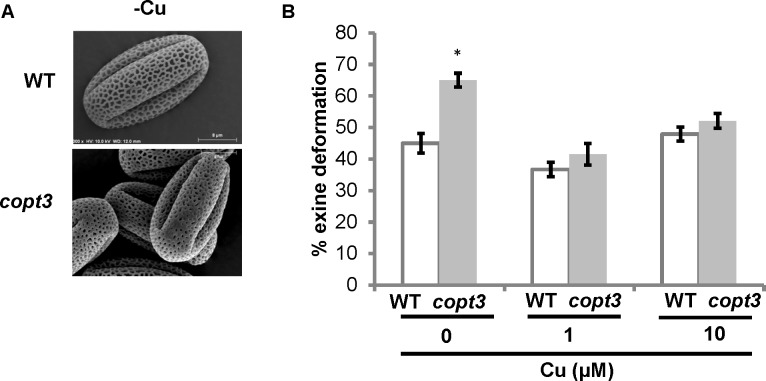
Pollen grains morphology of *copt3* knockout plants. **(A)** Electronic photography of representative wild-type (WT) and *copt3* pollen grains obtained from flowers at the anthesis stage. **(B)** Quantification of exine deformation in pollen grains. The plants were grown in soil under long day conditions and watered with Hoagland’ solution with 0 (–Cu), 1 and 10 μM CuSO_4_. Bars correspond to means ± standard deviation of five biological replicates of 15 flowers. One hundred pollen grains were analyzed for each genotype and condition. Asterisks indicate statistical differences (*P* < 0.05) according to *Z*-test.

In agreement with the observation that *COPT3* expression was higher in pollen, the *copt3* mutant showed a significantly higher percentage of pollen ornamentation defects than the WT plants, but only under Cu deficient conditions (**Figure 8**). *TCP16* was also highly expressed in pollen and abortion of early pollen development has been shown in the *TCP16RNAi* plants (Takeda et al., 2006). These results reinforced the relevance of Cu homeostasis for pollen viability and the temporal and reciprocal regulation established between TCP16 and COPT3, necessary to fully accomplish this crucial process in plants.

## Discussion

COPT1 and COPT3 transporters belong to two subfamilies (pmCOPT and imCOPT, respectively) involved in Cu^+^ uptake from different extra- and intra-cellular pools. The *COPT1* and *COPT3* are two flanking genes organized head-to-head in opposite orientations. In general, bidirectional activity was shown to be an inherent feature of most promoters, being especially relevant in divergent promoters (Seila et al., 2008; Wakano et al., 2012). In *Arabidopsis*, 5,763 divergent gene pairs were reported (Krom and Ramakrishna, 2008) and among them, 462 are separated by a small distance (<250 bp) sharing a single bidirectional promoter that may regulate the co-expression of the two genes (Dhadi et al., 2009). The intergenic region between transcriptional start sites of *COPT1* and *COPT3* is 2,266 bp (Supplementary Figure S1A), a similar size to the 2,177 DNA segment between the genes *cab1* and *cab2* which was shown to function as a bidirectional promoter (Mitra et al., 2009). It is thus possible, that *COPT1* and *COPT3* spatial expression is co-regulated via the shared bidirectional promoter. Indeed, both were mostly present in pollen, seeds and vascular bundles (**Figures 1B** and Supplementary Figure S3A) (Sancenón et al., 2004; Bock et al., 2006). However, whereas *COPT3* was expressed early in pollen development, *COPT1* was expressed at later stages (Bock et al., 2006). Indeed, Cu is highly required for pollen development and its regulated delivery through COPT transporters could be an important step. Both SPL7 and a Cu-DEFICIENCY-INDUCED TRANSCRIPTION FACTOR 1 (CITF1) belonging to the bHLH family (bHLH160) were recently shown to participate in the regulation of Cu delivery to the anthers and in jasmonic acid synthesis during Cu deficiency (Yan et al., 2017).

**FIGURE 9:**
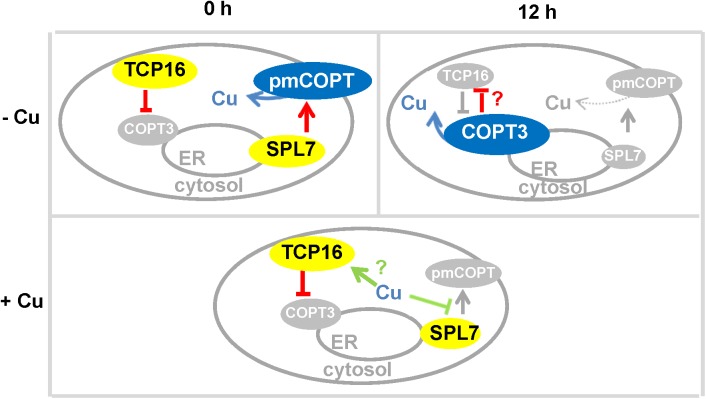
Model for TCP16 function in copper homeostasis. Scheme of the temporal (0 and 12 h) regulation of *pmCOPTs* and *imCOPT3* expression by *TCP16* and *SPL7* in wild-type seedlings under different Cu status (–Cu and +Cu).

Our data indicated that COPT3 might be located in a compartment of the secretory pathway, which could be the ER, where COPT3 would recover Cu^+^ from the ER lumen. Although further experimental approaches are required to localize COPT3 to a precise organelle, it is interesting to note that the ER was also the proposed location of the Cu deficiency sensor SPL7 (Garcia-Molina et al., 2014). SPL7 was proposed to sense both cytosolic and the ER lumen Cu status. Our COPT3 localization data pointed that COPT3 could be interestingly involved in the partitioning of these two differential Cu pools (**Figure 9**).

The fact that the *COPT3* transcriptional expression pattern was initially described as not being affected by Cu status in the medium (Sancenón et al., 2003), could be attributed to the particular temporal dependence of the Cu-regulation of *COPT3* expression. Both *COPT1* and *COPT3* present several GTAC elements nearby the translational start sites that could be involved in SPL7-mediated Cu deficiency responses (Yamasaki et al., 2009; Bernal et al., 2012). In regard to this, the expression in phase of both the activator *SPL7* and the target *COPT1* could drive a robust Cu deficiency response for *COPT1* expression, whereas the antiphase expression between the SPL7 and the target *COPT3* could temporally affect the intensity of the SPL7-mediated regulation, as already modeled (Peñarrubia et al., 2015). This result suggested that, at the temporal level, the promoter region could be alternatively used in each direction for *COPT1* and *COPT3* transcription, instead of co-expression. However, the SPL7-mediated antiphase regulation of *COPT3* expression cannot explain *per se* the anti-correlated expression observed at the temporal level between *COPT1* and *COPT3* driven from a putative bidirectional promoter (**Figure 1C**), but rather suggested the presence of other temporal transcriptional regulators.

Thanks to the TRANSPLANTA consortium, we could screen for conditionally inducible TFs that might participate in the response to Cu availability (Coego et al., 2014). The TCP family was one of the most overrepresented with regard to the number of positive members in the screen. Moreover, the presence of putative elements denoted CAREs in the *COPT3* and *COPT5* promoters, as well as in other Cu homeostasis components (Supplementary Table SII), is compatible with a regulatory role of TCPs in Cu homeostasis. We selected the TCP family for further study, because pollen morphology was also affected in plants with altered levels of *COPT1*, *COPT3*, and *TCP16* (Sancenón et al., 2004; Takeda et al., 2006; this study). Moreover, CHE is a TCP factor that was involved in *CCA1* repression by interacting with TOC1, both components of the circadian clock (Pruneda-Paz et al., 2009), which expression was altered in *COPT1* overexpressing plants (Andrés-Colás et al., 2010). *COPT1* and *COPT3* overexpression displayed similar phenotypes that were attributed to the temporal deregulation of the Cu entrance that could affect the circadian rhythms (Andrés-Colás et al., 2010). This fact could explain the phenotype observed for the *COPT3* overexpressing and *copt3* seedlings under Cu deficiency in this work. Finally, in microarrays analysis performed in transgenic plants with modified levels of *COPT2*, differential expression of several TCP members was observed (Perea-García et al., 2013).

There are several lines of evidence to support the model that TCP16 represses *COPT3* expression. First, TCP16 specifically bound to the *COPT3* and *COPT5* promoters *in vitro* as shown by EMSA (**Figure 3**). And second, *COPT3* was repressed as *TCP16* expression increased (**Figure 4**) and conversely, *COPT3* was upregulated in a *tcp16* mutant (**Figure 6**). The presence of a putative TCP16 binding site nearby the translational start site of *COPT3,* but not of *COPT1*, could account for the particular temporal repression of *COPT3*. The relative short distance (109 bp) between the GTAC boxes and the putative TCP16 binding site in the *COPT3* promoter (Supplementary Figure S1) brings to discussion if there is a competence between SPL7 and TCP16 for binding. Any kind of interaction between the activation and the repression function of SPL7 and TCP16, respectively, is also plausible since TCPs interact with a wide variety of other TF families, including other SPL member in the described TCP4-SPL9 temporal interaction taking place during flower development (Rubio-Somoza et al., 2014; Bemer et al., 2017). Furthermore, as a conclusion of the *COPT3* expression analysis, TCP16 could act as a repressor of COPT3-mediated Cu transport in a time specific manner.

Under Cu deficient conditions, we hypothesized that COPT3-mediated Cu recovery from the secretory pathway was repressed by TCP16 at 0 h, while the pmCOPT would participate in the uptake extracellular Cu at this time being activated by SPL7 (**Figure 9**). Under Cu excess, *pmCOPTs* were not activated by SPL7 and TCP16 will further repressed *COPT3* and *COPT5* along day and night (**Figure 9**). On the other hand, the expression of the *pmCOPT* transporters at 12 h could be subjected to a feedback autoregulatory loop that was proposed to act as a biochemical oscillator (Peñarrubia et al., 2010). Moreover, COPT3 and TCP16 were mutually repressing each other’s expression. TCP16 may directly act as a *COPT3* repressor, whereas COPT3 probably indirectly affected *TCP16* expression. Subsequently, Cu entrance from extracellular pools was prioritized at dawn and Cu mobilization from internal pools was favored at dusk. Whether this affected cytosolic Cu or the destiny of Cu coming from the different pools was being used for separate purposes still deserves further investigation. In addition, *HMA5* and *RAN1* also display putative CARE elements in their promoters (Supplementary Table SII) that could indicate that Cu transport in both directions (entrance and exit) through internal membranes was under control of TCPs.

The fine regulation exerted by TCP16 as a repressor of Cu entrance from internal stores specifically at dawn suggested a temporal division requirement for incompatible processes. Among the possibilities, the avoidance of a putative excessive increase in oxidative stress in this period that could not be properly counteracted. In this sense, Cu^+^ uptake through COPT transporters imposed an increased oxidative stress (Rodrigo-Moreno et al., 2013) that could interfere at multiple cellular processes and damage biological structures (Ravet and Pilon, 2013). The redox state of the cell was shown to influence the DNA binding ability of class I TCP proteins (Viola et al., 2013). The oxidation of a conserved cysteine residue (C-20) leaded to the formation of intermolecular disulfide bonds that cannot bound target promoters (Viola et al., 2016). Although the C-20 residue is not conserved, a single cysteine (C-107) residue is present in TCP16 (Supplementary Figure S6A).

On the other hand, *TCP16* repression could be aimed to protect a Cu sensitive process operating at dawn. In this sense, a specially Cu sensitive process that takes place in the mitochondria is the Fe-S cluster assembly, required for multiple processes including the respiratory electron transfer chain (Brancaccio et al., 2017). Since the mitochondrial matrix contains a labile Cu^+^ pool and is also the place where the Fe-S cluster assembly machinery resides, a strict temporal regulated Cu uptake might prevent a blocking of mitochondrial Fe-S protein maturation (Brancaccio et al., 2017). This process is in agreement with the regulatory function of mitochondrial proteins by TCPs (Welchen and Gonzalez, 2006) and is also connecting Fe and Cu homeostasis, as already described for the *copt2* mutant (Perea-García et al., 2013). Accordingly, *tcp16* seedlings were sensitive to Cu excess (**Figure 6**), maybe due to an impaired temporal Cu entrance to the mitochondria. The source of Cu that reaches organelles from an endosymbiotic origin, such as mitochondria and chloroplasts, remains an unsolved question. Under environmental nutrient deprivation, a putative Cu source is the lumen of the endocytic compartments that were recently shown to participate in dynamic intracellular metal homeostasis (Blaby-Haas and Merchant, 2014; Hong-Hermesdorf et al., 2014). Although further work is needed to confirm this hypothesis, the internal membrane COPT3 and COPT5 transporters might participate in Cu delivery from the secretory pathway to organelles under metal deficiency. In agreement, photosynthesis is affected in *copt5* mutants (Garcia-Molina et al., 2011). The mitochondrial *SDH1-2* promoter displayed 3 putative CARE elements (Welchen and Gonzalez, 2006) and it was shown to be regulated by TCPs (Giraud et al., 2010). Moreover, *SDH1-2* was a good marker for mild Cu excess (Andrés-Colás et al., 2013). In accordance, *SDH1-2* expression was down-regulated in the *copt3* mutant (**Figure 7B**). This result suggested that a Cu-TCP interplay may mediate mitochondrial *SDH1-2* expression. This would constitute a new pathway for gene expression regulation under mild Cu excess that might be aimed to protect mitochondria from Cu toxicity.

The fact that *COPT1* and *COPT3* were mostly expressed in vascular tissues points to a role for the temporal differences in metal long distance transport. In this sense, since the higher Cu affinity for common metal chelators (Álvarez-Fernández et al., 2014), competition with other metals, such as Fe, in the xylem transport could involve a metal interference in long distance transport, especially relevant under metal deficiencies. Although further work will be needed to address this hypothesis, a putative solution could be a metal differential temporal arrangement in vascular transport.

Finally, the *in silico* analysis of the hormone-responsive *cis*-elements present in the promoter sequences (1,000 bp upstream of the five prime untranslated region) from the *COPT1* and *COPT3* genes indicated a differential hormonal response (Peñarrubia et al., 2015). Major differences were observed for those *cis*-elements involved in ABA and gibberellic acid (GA) signaling. Whereas the total elements for ABA were 11 and 3, those for GA were 7 and 20 in the *COPT1* and *COPT3* promoters, respectively (Peñarrubia et al., 2015). Since the antagonism between ABA and GA is well-known (Weiss and Ori, 2007), as well as their interplay with the circadian clock (Atamian and Harmer, 2016) and their wide crosstalk with TCPs (Nicolas and Cubas, 2016), the results shown here underscore the role of phytohormones in the temporal orchestration of metal homeostasis that might control plant development depending on the environmental nutrient conditions.

## Author Contributions

LP conceived the idea and wrote the manuscript. MP, NA-C, and SA-G conceived and performed the COPT3 localization experiments. NA-C performed the TF screening. NA-C and AC-S performed the physiological and molecular experiments in mutant plants.

## Conflict of Interest Statement

The authors declare that the research was conducted in the absence of any commercial or financial relationships that could be construed as a potential conflict of interest.

## Footnotes

1 https://imagej.nih.gov/ij/

2 www.arabidopsis.org

3 https://psort.hgc.jp/

4 https://genevestigator.com/gv/doc/intro_plant.jsp

5 http://diurnal.mocklerlab.org/
