# Supplementary material for: Expression of the Intracellular COPT3-Mediated Cu Transport Is Temporally Regulated by the TCP16 Transcription Factor
Source: Front Plant Sci. 2018 Jul 3;9:910. doi: 10.3389/fpls.2018.00910 (PMC6037871; doi:10.3389/fpls.2018.00910)
Supplement: Supplementary file 1 [file Presentation_1.pdf]

A

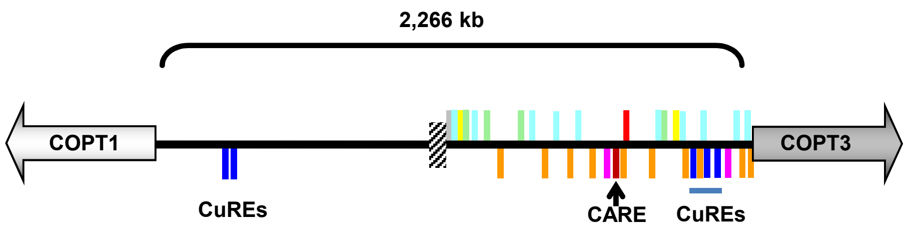

B

```
-1351 CATAATGTTG ATGTGTTATT ATAAGGTTGG TTTGTTGAAT GATAACATTA ATAATGAACC
-1291 GCTAAAATAC GGCCTTTTAC ATGTTCTTCA TTCAAGGCTT AGTTTACAAA AAGTTTTATA
-1231 ATATTTATCG ATTGGACTAT TGTTTCCGTG TTGAAAAATC TAACGGCTAC ATAGTAGTTT
-1171 TATTTGTCAT ATCAAATACT GAAGCATGTC AATATGTCAT CACTGTCCAT AATTCTTGTT
-1111 TCTTGGATTT TCTTAATAAA TTATTTGTTT CCTTTCACAA ACAGTATTGT TTCATAGACA
-1051 AATTGTATGT TCTGTCAGTT AGCATGAAAA ATTGTATGTT AGTCTACAAA ATAAATCTAA
 -991 TAGATTCAAA CATTATAGTT TACTAAAAAA AAAGATAAAG AAAAACATAG GAGCTTTGCC
 -931 AATTATTGAA GCAGAGATAT CTGATTGATT GATTAGTTTA AAACTTAACT ACGATTGAAT
 -871 AAAGGAACAA CACAAAAGAT ATCGATGACG ACCAGATACC ACAAGAAAAG AAAAGATCAA
 -811 GAAAGAAAGA AGAAGATATT TGATGTGAGG TCATAAAAGA GACGTATTAT TATATGGACT
 -751 TTCCAAAAAT ACATCGTAAA CATTACGTTG ACGAAACTCA TTCAGGCGAA CGATTACGTC
 -691 TGACACCGTT CGAGGGTTTA TTGGCATTTA TTTACTTTAT CCATGATAAA ATCTAAATTG
 -631 AGTAAATTGA ATTTTGGCCT GTGAGGTTAA TAATGATTAA GAAGGTAATT GTATGTTGTT
 -571 GTTTTTTTTT TACTATGGAA CGGATAACAA AAAAAATATT GGGCTTGTTT AAAGAAATTG
 -511 TCAATGACTC TGGATTCTTT TTTTCTCAGT CCATGGCATA AAAATACCAG TGTTATTAAA
 -451 AAAATTGAGC CCATAACAAA GCTGTGTTTT TTTTGAACAC GCCCATAACA AAACTTTCTA
 -391 ATTACCATTT TACGACCATA TTAATAACAA ATATTAGGAT TTTTTTTTTT AATTTGCTTT
 -331 TAACAGTCAA GCTAGAAACC AGACGAAATC ACAGTCTTTA GTCAAAAACA ATATAAAAAA
 -271 AAAGATTCTA GATTTTTTCA GCTTTGGATA ATTTACTTTT TCAATTTTAA GACTTAAAAG
 -211 GAACAGATCT TTGGACATAA TCTCAACAAA ATATCTTTGA AGTACACTTT TAAACTCTAA
 -151 TAAAATCACA ATTATCGCAT TATTGTTGTA AAAATGTACA TTGTACTACA CACCAAACTC
  -91 CAACCCAAAA ACAAAACCCT TATAAATACA CACACACAAG TATACACAAC AACAAAATCT
  -31 TCACATTGCA CGTAAAAAAT ATCTTTATAC AATG
```

C

```
MNGMSGSSPAAPAPSPSSFFQHRHRHGGMMHMTFFWGKTTEVLFDGWPGTSLKMYWVCLAVIFVIS
AFSECLSRCGFMKSGPASLGGGLLQTAVYTVRAALSYLVMLAVMSFNGGVFVAAMAGFGLGFMIFG
SRAFRATSSNSHTEVQSHC
```

Figure S1. Promoter region and protein sequence of the *COPT3* gene. (A) Scheme of the common promoter region between *COPT1* and *COPT3* genes. Genes are indicated with arrows and the different promoter motifs are represented in colors as indicated in panel B. **(B)** Motifs in *COPT3* promoter region. Numbers represent the relative position respect to the first nucleotide of the start codon, which is +1 and indicated with an arrow. The most relevant motifs are represented with colors: Orange, pollen expression boxes; pink, embryo expression boxes; green, endosperm expression boxes; yellow, aleurone expression boxes; red, plastid expression box; light blue, light response boxes; dark blue, Cu response boxes. The TATA box is indicated in bold letters, the CAAT box is underlined, and the TCP binding motif is in a box. **(C)** COPT3 protein sequence. The putative chloroplast target signal is underlined.

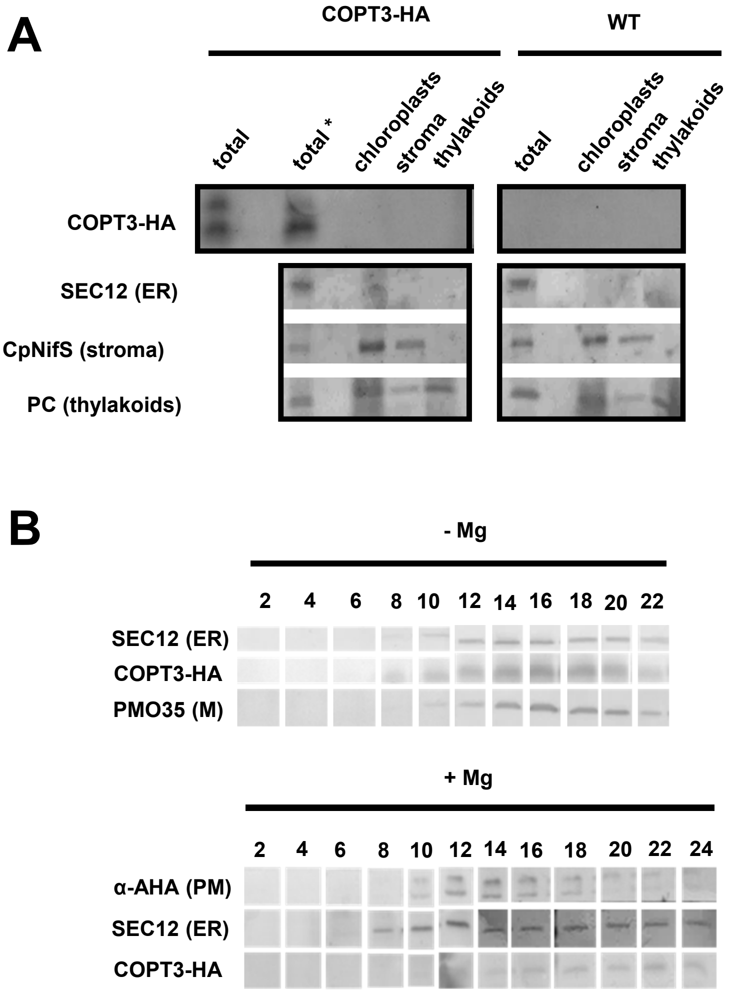

Figure S2. COPT3 subcellular localization in Arabidopsis protein fractions. (A) Extracts of total proteins, chloroplasts and stroma and thylakoids fractions from leaves of 3- to 4-week-old plants expressing the COPT3–HA fusion and grown in soil under long day conditions. The different fractions were electrophoresed, blotted, and immunodetected using antibodies against the HA epitope, the ER SEC12, the stroma CpNifS and the thylakoidal PC markers. Images from 1 preliminar experiment are shown. The asterisk indicated that the extract was kept in grinding buffer during the time of the whole isolation process. Whole blots are shown in panel C. **(B)** COPT3 subcellular localization by sucrose gradient density centrifugation. Protein extracts from the leaves of 4-week-old plants expressing the COPT3–HA fusion protein, and grown in soil under long day conditions, subjected to sucrose density gradient centrifugation with or without Mg. The different fractions were electrophoresed, blotted, and immunodetected using antibodies against the HA epitope, the ER SEC12, the plasma membrane α-AHA, and the mitochondrial PMO35 markers. Images from 1 preliminar experiment are shown. Whole blots are shown in panel D. COPT3-HA, 18 KDa; SEC12, 43 KDa; CpNifS, 43 KDa; PC, 13 Kda; α-AHA, 90-95 KDa; PMO35, 29 KDa.

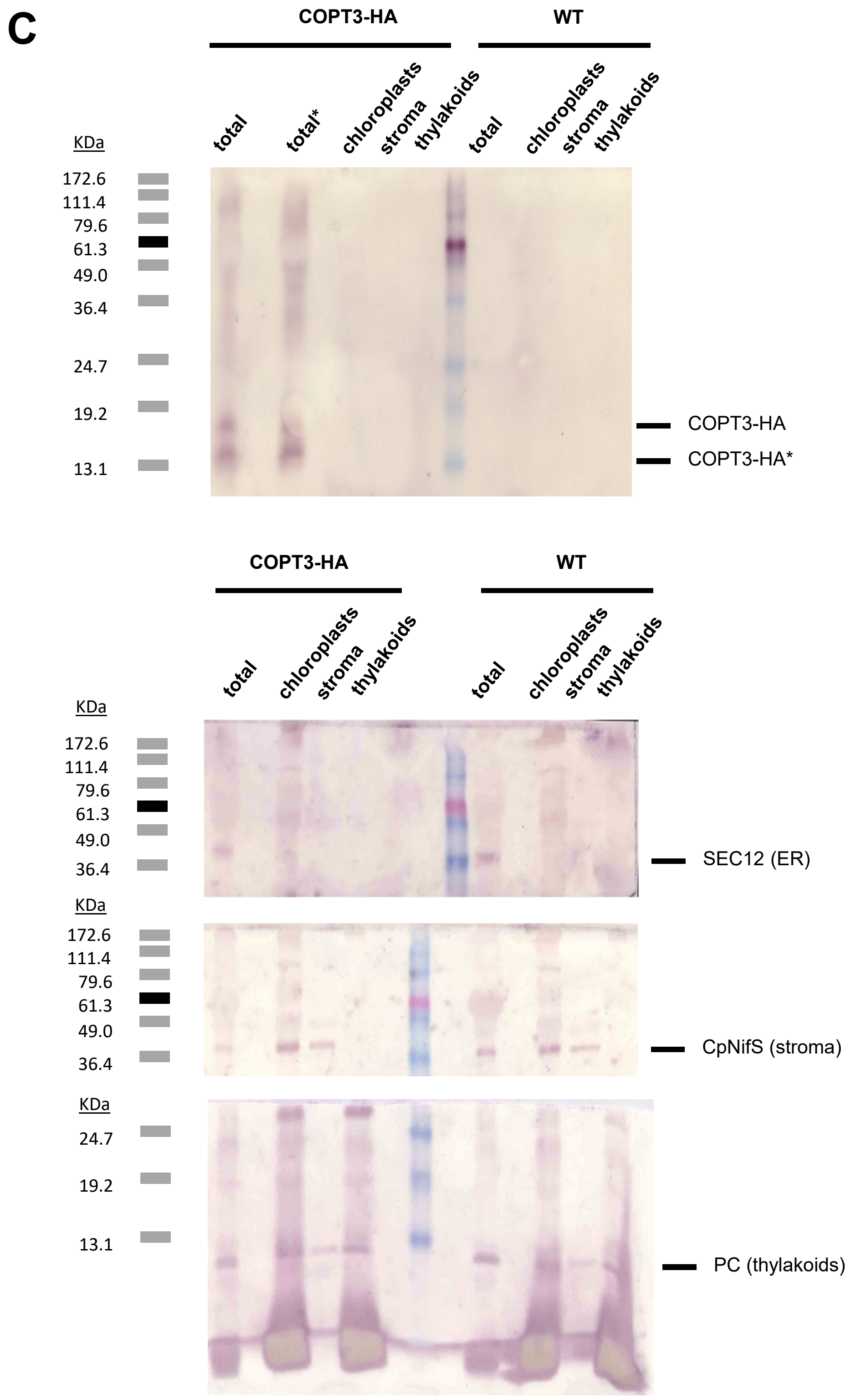

Figure S2 (continuation). COPT3 subcellular localization in Arabidopsis protein fractions. (C) Whole blots from panel A. *, putative degraded version of COPT3-HA

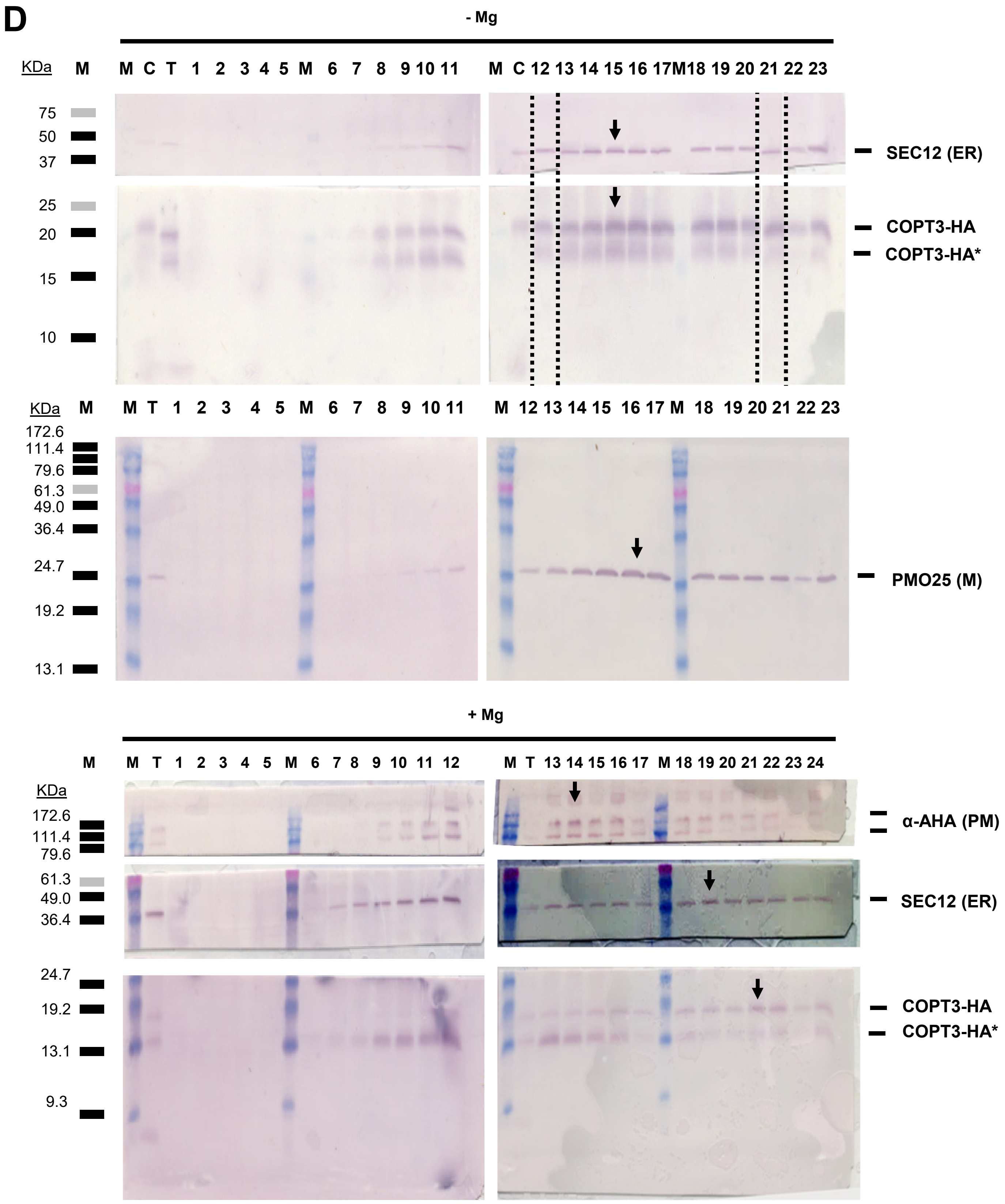

Figure S2 (continuation). COPT3 subcellular localization in Arabidopsis protein fractions. (D) Whole blots from panel B. *, putative degraded version of COPT3-HA M, protein ladder. C, previously analized sample of total protein extract from plants expressing the COPT3–HA fusion. T, total protein extract from the plants expressing the COPT3–HA fusion before its fraccionation. Arrows point to the more intense band in the fraccionation for each immunodetected protein.

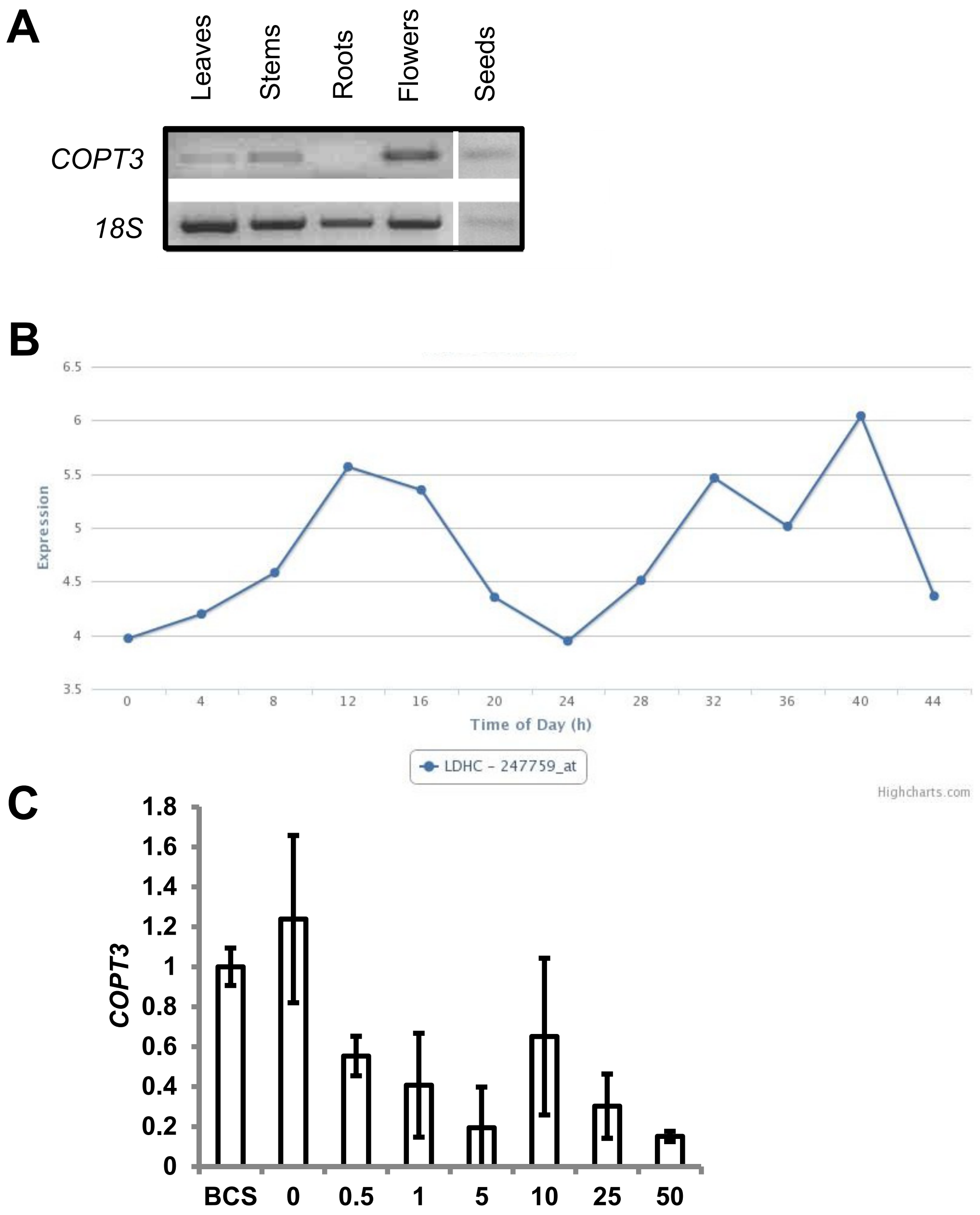

Figure S3. ***COPT3*** **expression pattern and regulation by Cu. (A)** *COPT3* expression in different tissues. Samples were taken from leaves, stem, roots, flowers and dried seeds of wild-type adult plants grown in soil under long day conditions. Total RNA was extracted and analyzed by RT-PCR with specific oligonucleotides for *COPT3*. The *18S* gene was used as loading control. Representative images from at least 2 biological samples are shown. **(B)** DIURNAL DataBase pattern of *COPT3* expression under 12 h light/ 12 h dark neutral photoperiod cycle (*http://diurnal.mocklerlab.org/*) (Mockler et al., 2007). **(C)** Expression of *COPT3* under different Cu status. 6-day-old wild-type seedlings grown in ½ MS medium (MS) and the same medium supplemented with 100 µM BCS (BCS) or 0, 0.5, 1, 5, 10, 25 and 50 µM $CuSO_4$. Samples were taken at 4 h under long day conditions. Total RNA was extracted and analyzed by RT-qPCR with specific oligonucleotides for *COPT3*. The relative expression in arbitrary units is represented. Values correspond to arithmetic means ($2^{-\Delta\Delta Ct}$) ± standard deviation from 3 biological replicates (*n*=3).

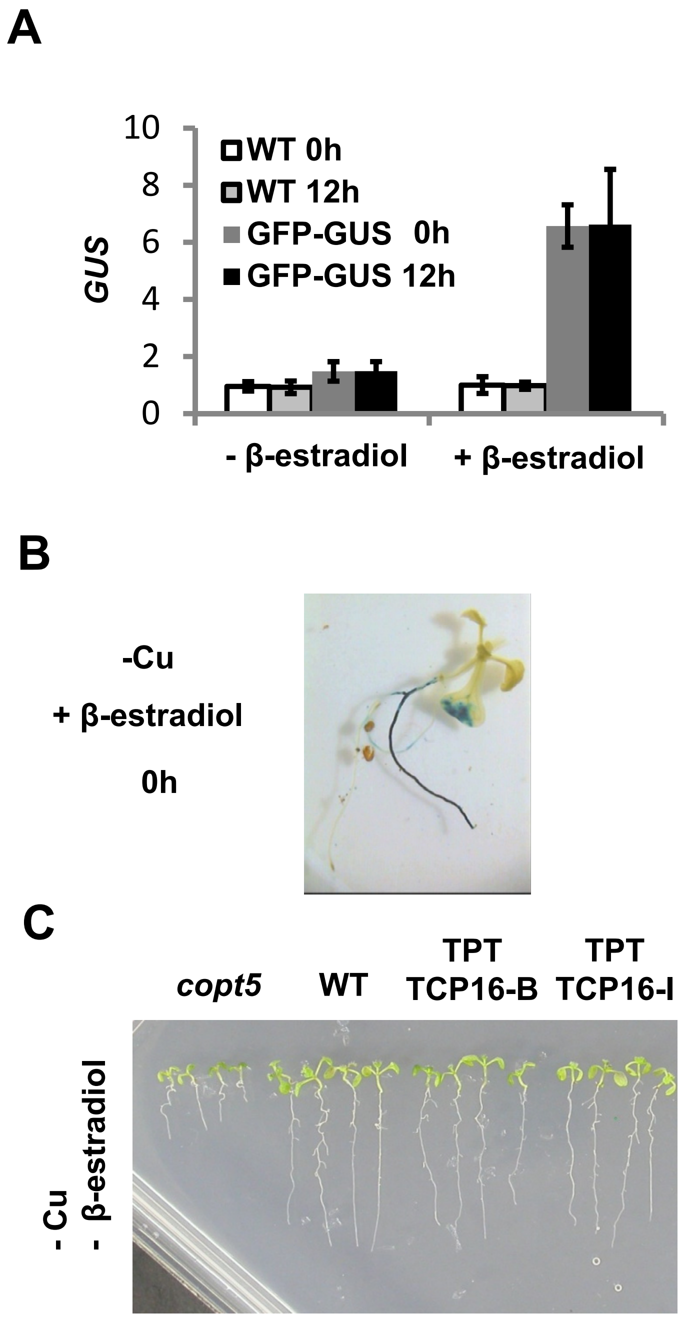

Figure S4. Short-term kinetics of *GUS* expression by β-estradiol. (A) 6-day-old wild-type (WT) and *ER8GWp:GUS-GFP* (GFP-GUS) seedlings grown in ½ MS medium supplemented with 100 µM BCS without (-β-estradiol) or with 2 µM β-estradiol (+β-estradiol). Samples were taken at 0 and 12 h of the 12 h light / 12 h dark cycle. Total RNA was extracted and analyzed by RT-qPCR with specific oligonucleotides for *GUS*. Values correspond to arithmetic means ($2^{-\Delta\Delta Ct}$) ± standard deviation from 3 biological replicate with 3 technical replicates for each (n=3). **(B)** Photograph of a representative 6-day-old *pER8GW:GUS-GFP* seedlings grown in ½ MS medium supplemented with 100 µM BCS (-Cu) and 2 µM β-estradiol (+β-estradiol), after GUS staining. A representative image from 10 seedlings is shown. **(C)** Photograph of representative 8-day-old *copt5*, wild-type (WT), TCP16-B and TCP16-I TPT seedlings grown in ½ MS medium supplemented with 100 µM BCS (-Cu) without (-β-estradiol). A representative image of at least 2 independent experiments is shown.

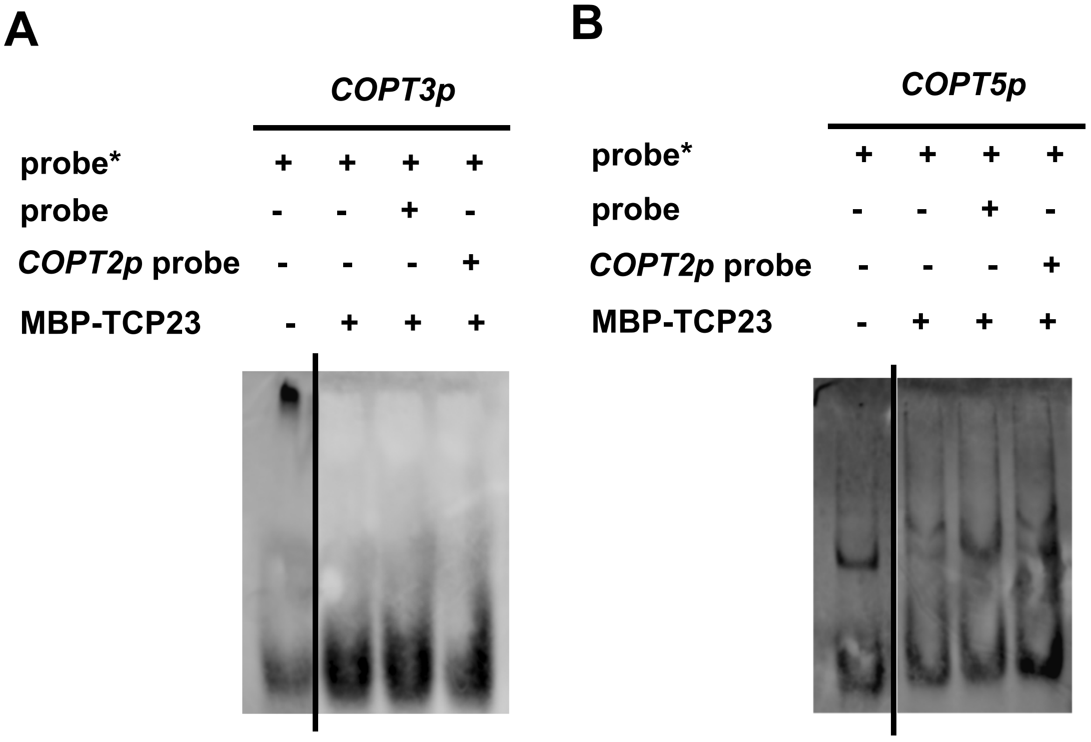

Figure S5. EMSA binding assay between TCP23 protein and *COPTs* promoters. Purified TCP23 protein was incubated with biotin-labeled (*) DNA probe from *COPT3* promoter **(A)** and *COPT5* promoter **(B)**, containing TCP box. Unlabeled probes were added as a competitor as indicated. Representative images of at least 2 independent experiments are shown.

A

```
MDSKNGINNS QKARRTPKDR HLKIGGRDRR IRIPPSVAPQ LFRLTKELGF
KTDGETVSWL LQNAEPAIFA ATGHGVTTTS NEDIQPNRNF PSYTFNGDNI
SNNVFFCTVV NTGHRQMVFP VSTMTDHAPS TNYSTISDNY NSTFNGNATA
SDTTSAATTT ATTTV
```

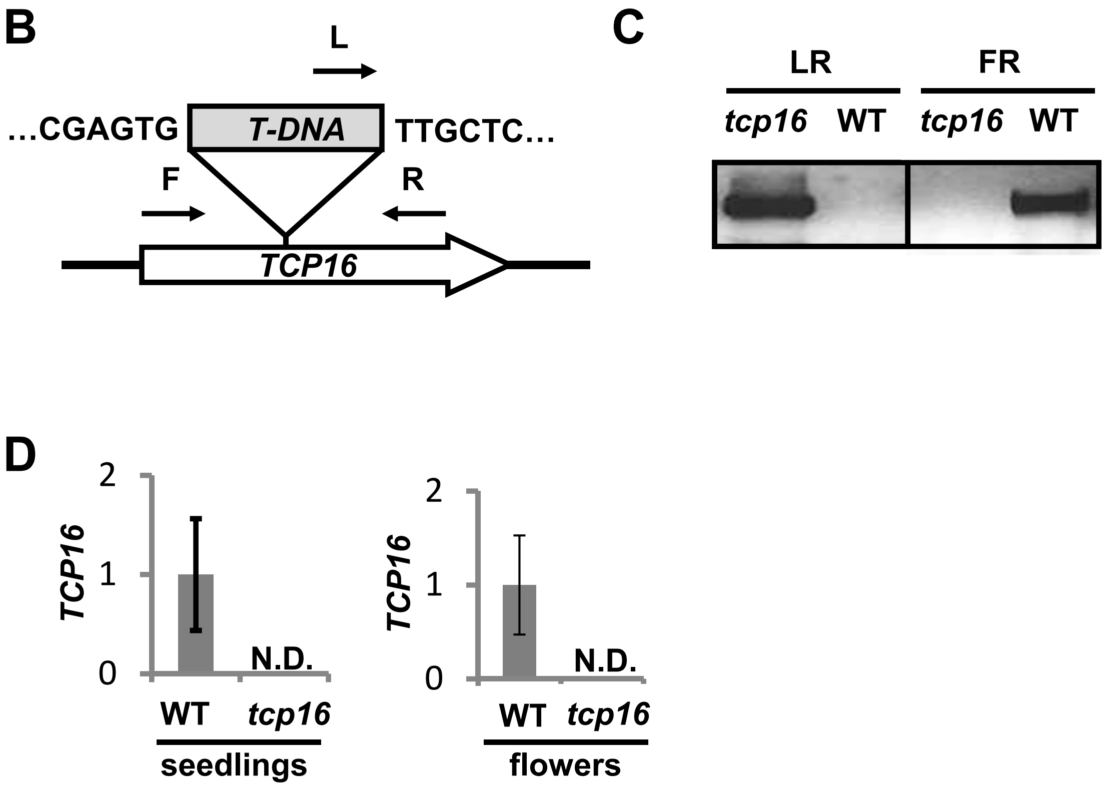

Figure S6. Selection of the *tcp16* knockout line. (A) Protein sequence. A putative Cu-binding Cys is indicated with a red circle. **(B)** Scheme showing the T-DNA insertion site in the knockout lines, and the position of the oligonucleotides used for PCR genotyping in panel B. **(C)** Genotyping of the knockout lines. Genomic DNA from the wild-type (WT) and the knockout seedlings was obtained and used for the PCR analysis. **(D)** 6-day-old wild-type (WT) and knockout seedlings grown in ½ MS medium supplemented with 100 µM BCS and flowers from adult plants grown in liquid ½ MS medium supplemented with 100 µM BCS. Samples were taken at 0 h (dark bars) of the 12 h light / 12 h dark cycle. Total RNA was extracted and analyzed by RT-qPCR with specific oligonucleotides for *TCP16*. The relative expression in arbitrary units is represented. Values correspond to arithmetic means ($2^{-\Delta\Delta Ct}$) ± standard deviation from at least 2 biological replicates ($n \geq 2$). N.D., not detectable.

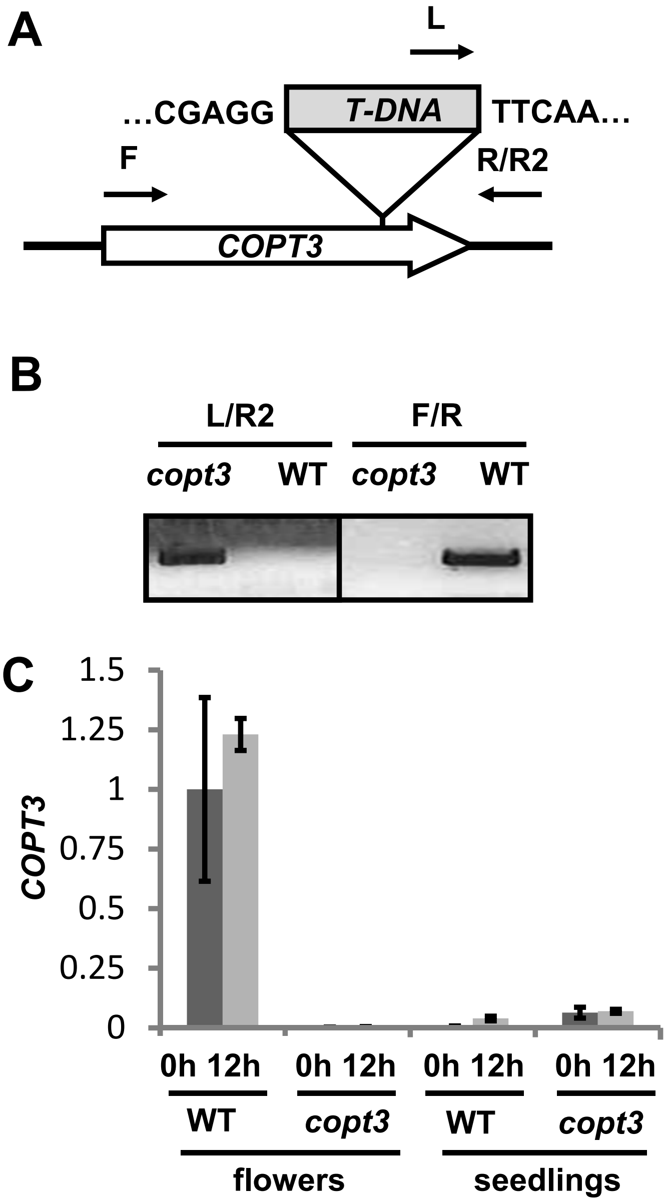

Figure S7. Selection of the *copt3* knockout line. (A) Scheme showing the T-DNA insertion site in the knockout lines, and the position of the oligonucleotides used for PCR genotyping in panel B. **(B)** Genotyping of the knockout lines. Genomic DNA from the wild-type (WT) and the knockout seedlings was obtained and used for the PCR analysis. **(C)** 6-day-old wild-type (WT) and knockout seedlings grown in ½ MS medium supplemented with 100 µM BCS and flowers from plants grown in soil. Samples were taken at 0 h (dark bars) and 12 h (light bars) of the 12 h light/ 12 h dark cycle. Total RNA was extracted and analyzed by RT-qPCR with specific oligonucleotides for *COPT3*. The relative expression in arbitrary units is represented. Values correspond to arithmetic means ($2^{-\Delta\Delta Ct}$) $\pm$ standard deviation from at least 3 biological replicates ($n \geq 3$).

Table SI. Transgenic lines used in this work. The name of the line, the code and the reference where created are indicated.

Line	Code	References
TPT TCP16-B	3.45150.1B	Coego *et al*., 2014
TPT TCP16-I	3.45150.1I	Coego *et al*., 2014
GFP-GUS	-	Coego *et al*., 2014
copt5	-	García-Molina et al., 2011
TCP16 RNAi	-	Takeda et al., 2005
tcp16	N462818	This work
copt3	GK633G06	This work
COPT3-HA	-	Andrés-Colás et al., 2010
COPT3p-GUS	-	This work

Table SII. Cu homeostasis factors with TCP16 binding motifs. Number of TCP16 CAREs in Cu homeostasis factors. TCP16 other versions, indicate the presence of different versions of the TCP16 CAREs as indicated. CuRE, number of GTAC motifs in the upstream 500 bp in both strains. Cu Reg, median log_2 ratio values at high (10 μM) vs low (MS) Cu levels obtained in microarray analysis (Andrés-Colás et al 2013).

Factor	MIPS code	TCP16 GTGGNCCC	TCP16 other versions	CuRE	Cu Reg
COPT3	AT5G59040	0	TTGAGCCCAT	3	---
COPT5	AT5G20650	0	GTAAGCCCAC<br>GTGAGCCCAC	0	-0.014
HMA5	AT1G63440	0	ATCGGCCCAC	0	0.826
APX1	AT1G07890	1	0	0	-0.242
Diamine oxidase	AT1G31670	1	0	0	---
SPL1	AT2G47070	1	0	2	-0.039
SPL12	AT3G60030	1	0	1	0.124
Blue copper prot.	AT5G14345	1	0		---

Table SIII. Oligonucleotides used for EMSAs.

Name	Sequence
COPT3 F	TAAAAAAATTGAGCCCATAACAAAGC-BIOTIN
COPT3 R	GCTTTGTTATGGGCTCAATTTTTTTA-BIOTIN
COPT5 F	GTGTTATTGTAAGCCCACTGGACTATAATGTGAGCCCACGAAGAAAC-BIOTIN
COPT5 R	GTTTCTTCGTGGGCTCACATTATAGTCCAGTGGGCTTACAATAACAC-BIOTIN
COPT2 F	TCACAATAAATACGAACCGATTCTCT
COPT2 R	AGAGAATCGGTTCGTATTTATTGTGA

Table SIV. Oligonucleotides used for regular PCRs.

Name	Sequence
COPT3 F	AATACACACACACAAGTATACACAACAAC (C3 II)
COPT3 R	CCTAATCATTATTTCAACGGGAAACAAGG (C3 II)
COPT3 R2	AGAGAATTTAGATCGGAACGAACA (C3 I)
GKATseq	ATATTGACCATCATACTCATTGC (C3 I)
GKAT-PCR	CCCATTTGGACGTGAATGTAGACAC
TCP16 F	CGAAAAATGGAATTAACAACAGC
TCP16 R	CAACCGTACAAGGGAAAACG
18S F	TGGGATATCCTGCCAGTAGTCAT
18S R	CTGGATCCAATTACCAGACTCAA

Table SV. Oligonucleotides used for real-time PCRs.

Name	Sequence
TCP16 F	ATGGTAATGCTACCGCCAGT
TCP16 R	CAAACTGTGGTTGTGGCTGT
COPT3 F	TATTACAGACTGCGGTTTAC
COPT3 R	CGAAGACTCCTCCATTGAAC
COPT3 F2	AACAGTCACACCGAGGTTCA
COPT3 R2	TCAACGGGAAACAAGGAAAATAAA
COPT1 F	TTGCAATTTTCCTCTCCTCCCAA
COPT1 R	ATGATGGTCGAGGCATT
COPT2 F	CCTTTCGTATTTGGTGATGCT
COPT2 R	AAACACCTGCGTTAAAGGAC
SDH1-2 F	GGTGCCTTCGAGTTGCGTCG
SDH1-2 R	CCCTGCCGAAGGAGGAGCTG
CAS F	TGCTTCATCGACCATGGATA
CAS R	CGGCGTAAGATCACCTTTGT
GUS F	TTTGAAGCCGATGTCACGCCGT
GUS R	ACAAACGGTGATACGTACACT
UBQ10 F	TAATCCCTGATGAATAAGTGTTCTAC
UBQ10 R	AAAACGAAGCGATGATAAAGAAG
